# Oxygen‐carrying semiconducting polymer nanoprodrugs induce sono‐pyroptosis for deep‐tissue tumor treatment

**DOI:** 10.1002/EXP.20230100

**Published:** 2024-02-19

**Authors:** Fengshuo Wang, Yongliang Fan, Yue Liu, Xiangxin Lou, Linawati Sutrisno, Shaojun Peng, Jingchao Li

**Affiliations:** ^1^ State Key Laboratory for Modification of Chemical Fibers and Polymer Materials College of Biological Science and Medical Engineering Donghua University Shanghai China; ^2^ Department of Cardiovascular Surgery Shanghai General Hospital Shanghai Jiao Tong University School of Medicine Shanghai China; ^3^ World Premier International (WPI) Research Center for Materials Nanoarchitectonics (MANA) National Institute for Materials Science (NIMS) Tsukuba Japan; ^4^ Zhuhai Institute of Translational Medicine Zhuhai Precision Medical Center Zhuhai People's Hospital (Zhuhai hospital affiliated with Jinan University) Zhuhai Guangdong China

**Keywords:** deep tumors, prodrug, pyroptosis, semiconducting polymer, sonodynamic therapy

## Abstract

Sonodynamic therapy (SDT) has been explored for cancer therapy, especially for deep tumors due to its low tissue penetration restriction. The therapeutic efficacy of SDT is limited due to the complicated tumor microenvironment. This study reports the construction of oxygen‐carrying semiconducting polymer nanoprodrugs (OSPN_pro_) for deep tumor treatment via combining amplified SDT with pyroptosis. An oxygen carrier perfluorohexane, sonodynamic semiconducting polymer as the sonosensitizer, and reactive oxygen species (ROS)‐responsive prodrug are co‐loaded into a nanoparticle system, leading to the formation of these polymer nanoprodrugs. Such OSPN_pro_ show an effective accumulation in tumor tissues after systemic administration, in which they deliver oxygen to relieve tumor hypoxia microenvironment and thus mediate amplified SDT via producing ROS under ultrasound (US) irradiation, even when the tumors are covered with a 2‐cm chicken breast tissue. In addition, the ROS‐responsive prodrugs are activated by the generated ROS to trigger pyroptosis of tumor cells. Such a sono‐pyroptosis induces a strong antitumor immunity with obviously higher level infiltrations of effector immune cells into tumors. Therefore, OSPN_pro_‐based combinational therapy can greatly inhibit the growth of 2‐cm chicken breast tissue‐covered deep tumors and suppress tumor metastasis. This study offers a prodrug nanoplatform for treatment of deep tumor via sono‐pyroptosis strategy.

## Graphical abstract

An oxygen‐carrying semiconducting polymer nanoprodrug is reported to combine amplified sonodynamic therapy with pyroptosis for deep tumor treatment. The nanoprodrug delivers oxygen to relieve tumor hypoxia microenvironment and thus mediate prodrug activation for pyroptosis. This nanoprodrug‐based treatment greatly inhibits the growth of 2‐cm chicken breast tissue‐covered deep tumors and suppresses tumor metastasis.

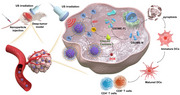

## 1 INTRODUCTION

Sonodynamic therapy (SDT) that combines sonosensitizers with ultrasound (US) to kill cancer cells through generating reactive oxygen species (ROS) has been explored as an attractive tactic for cancer therapy.^[^ ^1^ ^]^ Different from photodynamic therapy that relies on poorly penetrating light,^[^ ^2^ ^]^ SDT overcomes tissue penetration restriction because of the excellent tissue penetration depths of US, and thus has been used for treatment of deep tumors.^[^ ^3^ ^]^ In addition, SDT shows a high treatment selectivity by simply focusing US irradiation on tumor regions to reduce side effects.^[^ ^4^ ^]^ However, the therapeutic outcomes will be compromised by complicated tumor microenvironment.^[^ ^5^ ^]^ For example, the tumor hypoxia condition limits the ROS generating efficacies because of oxygen dependence for SDT.^[^ ^6^ ^]^ The high levels of antioxidants inside cancer cells will consume the generated ROS to reduce therapeutic efficacy.^[^ ^7^ ^]^ Sole SDT often fails to entirely remove tumors, especially for metastatic tumors. Therefore, it is highly necessary to overcome major obstacles of SDT for desired antitumor effect.

Pyroptosis is a specific cell death type regulated by gasdermin proteins,^[^ ^8^ ^]^ with membrane pore formations, cell swelling and releases of pro‐inflammatory cytokines, which is substantially different from traditional apoptosis.^[^ ^9^ ^]^ In addition to directly inducing cell death, pyroptosis can cause antitumor immunological effect by releasing the tumor antigens and inflammatory signaling molecules, which is important to reject remaining tumors and prevent tumor metastasis.^[^ ^10^ ^]^ Because the ROS levels are closely related to pyroptosis induction, some ROS‐generating therapies such as SDT can substantially improve pyroptosis efficiency.^[^ ^11^ ^]^ The combination of pyroptosis with SDT will offer an innovative modality for cancer treatments with the advantages of low therapeutic resistance and high antitumor effects.^[^ ^12^ ^]^ Currently, small molecular drugs have been used to initiate gasdermin‐dependent pyroptosis in diverse types of cancer.^[^ ^13^ ^]^ However, fast clearance and uncontrolled biodistribution of these drugs after systemic circulation greatly limit their bioavailability and therapeutic selectivity.^[^ ^14^ ^]^ More importantly, the uncontrolled initiation of pyroptosis in normal tissues will cause safety concerns.^[^ ^15^ ^]^

In view of different levels of biomarkers in tumor microenvironment and normal tissues, some tumor microenvironment‐responsive nanosystems have been proposed for regulating pyroptosis via achieving drug activation at tumor sites.^[^ ^16^ ^]^ For example, an acid‐activatable nanotuner was developed for spatiotemporally eliciting pyroptosis in early endosomes, but not in late endosomes/lysosomes.^[^ ^17^ ^]^ A ROS and glutathione (GSH) dual‐responsive nanoprodrug containing a chemotherapeutic (paclitaxel) and photosensitizer was constructed to control drug release in tumors for inducing pyroptosis‐based cancer immunotherapy.^[^ ^18^ ^]^ Although these nanosystems can regulate the activity of pyroptosis in tumor sites and potentially relieve off‐target side effects, the activation efficacy remains a great challenge due to the insufficient levels of biomarkers in tumor microenvironment.^[^ ^19^ ^]^ Alternative strategies should be explored to achieve pyroptosis‐based therapy in a safer and more effective manner.

**FIGURE 1:**
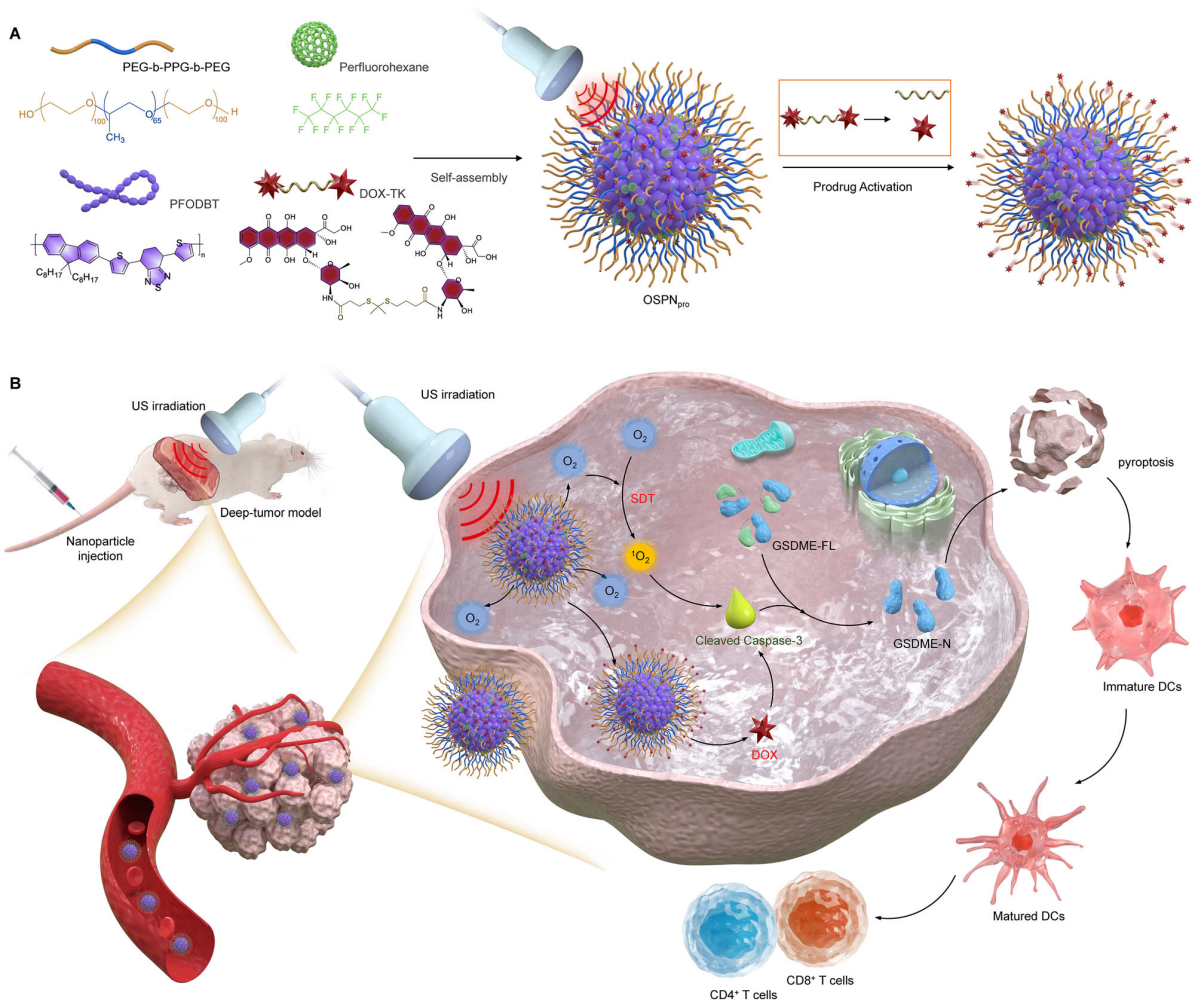
Design of oxygen‐carrying semiconducting polymer nanoprodrugs (OSPN_pro_) for treatment of deep‐tissue tumors via inducing sono‐pyroptosis. (A) Schematic illustration for the synthesis of OSPN_pro_. (B) Schematic illustration of prodrug activation and working mechanisms of OSPN_pro_ for treatment of deep‐tissue tumors via inducing sono‐pyroptosis.

Herein, we report oxygen‐carrying semiconducting polymer nanoprodrugs (OSPN_pro_) to combine amplified SDT with controlled pyroptosis for treatment of deep‐tissue tumors. OSPN_pro_ are formed via nanoprecipitation of a synthesized ROS‐responsive prodrug based on doxorubicin (DOX), an oxygen carrier perfluorohexane (PFH), a semiconducting polymer and a polymer matrix (Figure 1A). Semiconducting polymer with good biocompatibility have been explored for different types of cancer treatments, including photothermal therapy,^[^ ^20^ ^]^ photodynamic therapy^[^ ^21^ ^]^ and SDT.^[^ ^22^ ^]^ DOX is a chemotherapy drug that can efficiently trigger caspase‐3‐mediated pyroptosis.^[^ ^23^ ^]^ PFH carrying oxygen was delivered to tumor sites after the effective accumulation of OSPN_pro_, which relieved tumor hypoxia microenvironment. With ultrasound (US) irradiation, semiconducting polymers within OSPN_pro_ generated singlet oxygen (^1^O_2_) for SDT, and the SDT action was further enhanced due to the increased oxygen levels in tumor sites (Figure 1B). The ROS‐responsive prodrugs were then activated by the generated ^1^O_2_ to release active DOX, which worked as an inducer to trigger tumor cell pyroptosis, further leading to activation of a strong antitumor immunity. More interestingly, OSPN_pro_ could be used for treatment of 2‐cm chicken breast tissue‐covered deep tumors to achieve significant inhibition of tumor growth and suppression of tumor metastasis.

## 2 RESULTS AND DISCUSSION

### 2.1 Property analysis of nanoprodrugs

To construct OSPN_pro_, a ROS‐responsive prodrug was synthesized via conjugating DOX with a ROS‐responsive thioketal (TK) linker (Figure S1, Supporting information). The successful synthesis of ROS‐responsive DOX‐TK prodrug was confirmed (Figure S2, Supporting information). OSPN_pro_ were formed after nanoprecipitation using PFH, poly[2,7‐(9,9‐di‐octyl‐fluorene)‐alt‐4,7‐bis(thiophen‐2‐yl)benzo‐2,1,3‐thiadiazole] (PFODBT), and DOX‐TK. The nanoprecipitation of PFODBT and PFH resulted in the formation of control nanoparticles without prodrug loading (OSPN).

**FIGURE 2:**
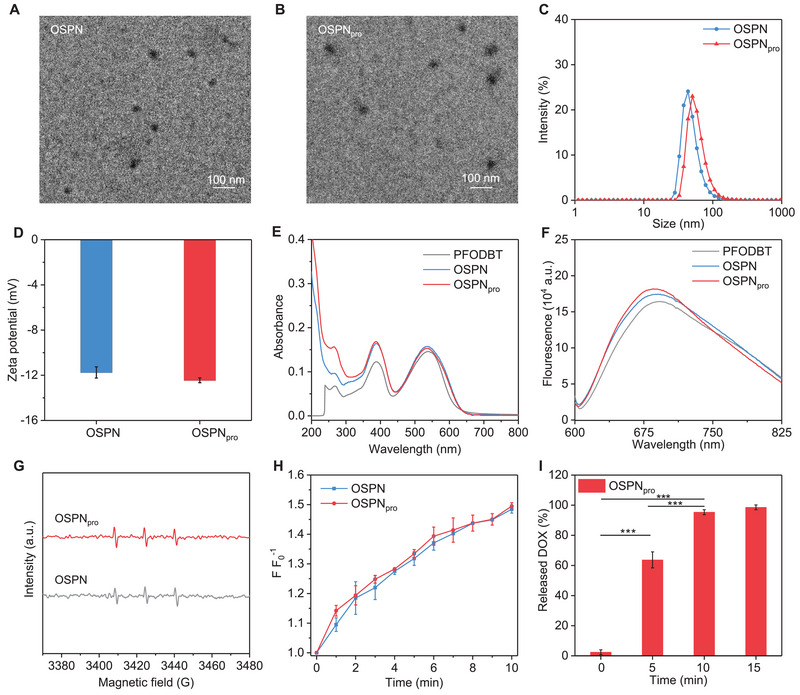
Characterization of nanoprodrugs. (A) The morphology of OSPN observed using TEM. (B) The morphology of OSPN_pro_ observed using TEM. (C) Hydrodynamic size of OSPN and OSPN_pro_. (D) Surface properties of OSPN and OSPN_pro_ (*n* = 3). (E) UV–vis absorbance spectra of PFODBT, OSPN and OSPN_pro_ at the same concentration. (F) Fluorescence spectra PFODBT, OSPN and OSPN_pro_. (G) ESR spectra of OSPN and OSPN_pro_ after treatment by US for ^1^O_2_ generation evaluation. (H) The evaluation of sonodynamic effect of OSPN and OSPN_pro_ by measuring the generation of ^1^O_2_ (*n* = 3). (I) The release percentages of free DOX from OSPN_pro_ solutions after US treatment for various time (*n* = 3). Mean ± SD are presented in data, two‐tailed unpaired t test, ***p < 0.001.

Similar spherical shape was observed in the transmission electron microscope (TEM) images of OSPN and OSPN_pro_ (Figure 2A,B). After the loading of prodrugs, the hydrodynamic size of OSPN_pro_ (50.7 nm) was larger than that of OSPN (43.8 nm) (Figure 2C), which was consistent to the diameters in TEM images. The surface properties of OSPN (−11.7 mV) and OSPN_pro_ (−12.4 mV) were similar (Figure 2D). Both OSPN and OSPN_pro_ showed a good colloidal stability for at least 14 days in different solutions (Figure S3, Supporting information). As observed in the UV–vis absorbance spectra, OSPN and OSPN_pro_ displayed two characteristic peaks at 328 and 540 nm (Figure 2E), which were similar to PFODBT. In addition, the fluorescence intensity of PFODBT, OSPN and OSPN_pro_ was almost consistent (Figure 2F). These verified the successful loading of PFODBT into nanoparticles, and PFODBT loading did not affect the absorbance and fluorescence properties. The hemolysis analysis showed that OSPN and OSPN_pro_ did not obviously damage red blood cells at the concentration ranging from 6.25 to 100 μg mL^−1^ as the percentages were below 5.0% (Figure S4, Supporting information).

The sonodynamic effect and prodrug activation under US irradiation were then confirmed. After irradiation by US, the characteristic peaks of ^1^O_2_ were similarly detected in the electron spin resonance (ESR) spectra of OSPN and OSPN_pro_ (Figure 2G). As observed in the fluorescence spectra of OSPN and OSPN_pro_ solution containing a ^1^O_2_ probe (singlet oxygen sensor green, SOSG), the fluorescence intensities at 480 nm gradually increased as the function of US irradiating time for both solutions (Figure S5, Supporting information), verifying the effective ^1^O_2_ generation under US treatment. After US treatment for 10 min, the intensity for OSPN and OSPN_pro_ similarly increased by 1.5‐fold (Figure 2H). These results verified that OSPN_pro_ had a similar sonodynamic ^1^O_2_ generation efficacy as OSPN. The activation of prodrugs under US treatment was verified. Nearly no free DOX could be detected in OSPN_pro_ solution without US treatment, while which was detected for US‐treated OSPN_pro_ solutions (Figure 2I). After US treatment for 5, 10, and 15 min, the release percentage of free DOX reached 63.7%, 95.3%, and 98.5%, respectively. The activation mechanism of nanoprodrugs was summarized as follows: OSPN_pro_ produced ^1^O_2_ under US treatment to destroy the ROS linkers in DOX‐TK prodrugs, thus enabling on‐demand release of free DOX.

### 2.2 In vitro therapeutic effect evaluation and mechanism studies

**FIGURE 3:**
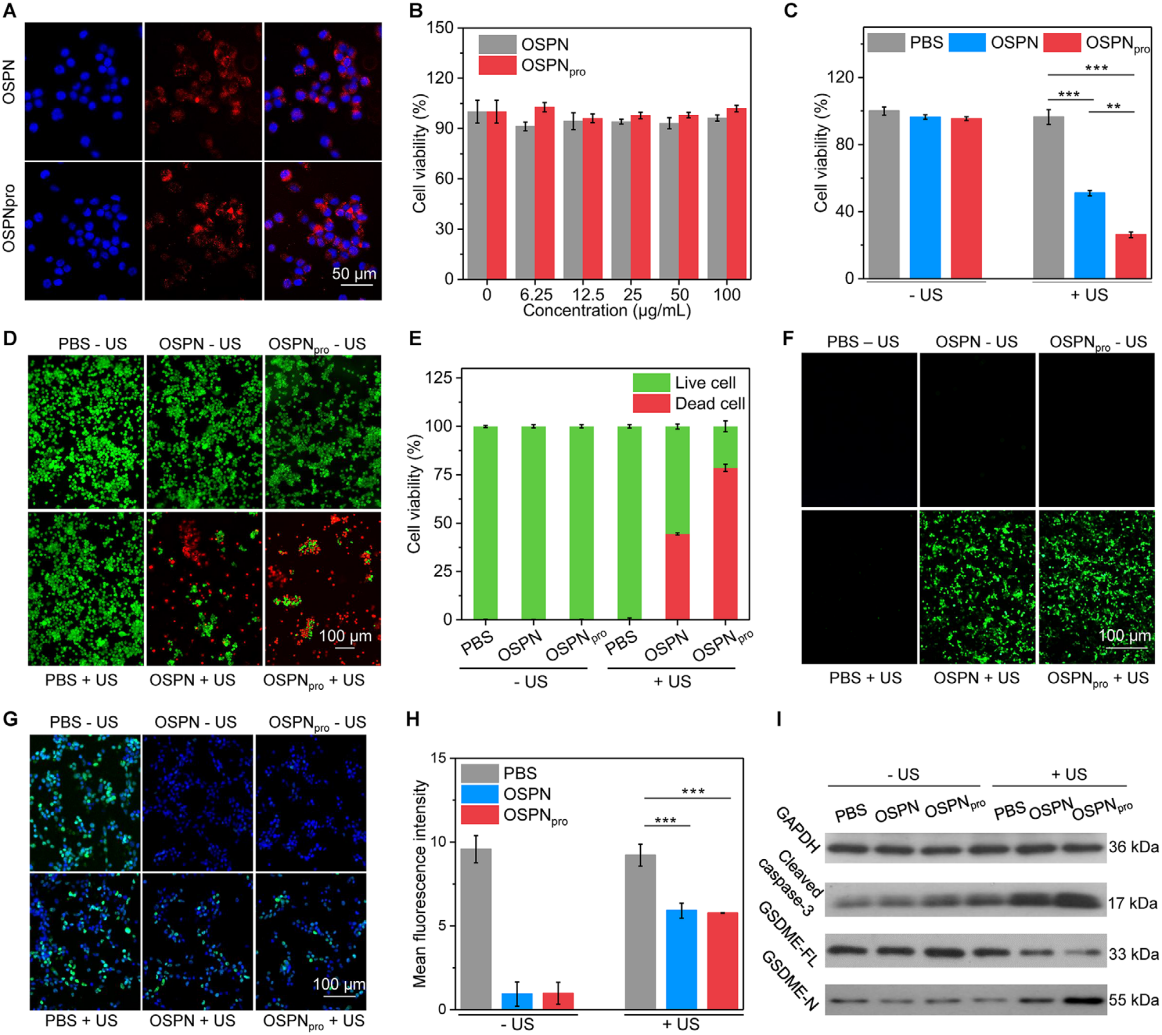
In vitro therapeutic effect evaluation and mechanism studies. (A) Cellular uptake analysis of OSPN and OSPN_pro_ by 4T1 cells using fluorescence imaging. (B) Analysis of cell viability of 4T1 cells (*n* = 5). (C) In vitro therapeutic efficacy after incubation with OSPN and OSPN_pro_ plus US treatment (*n* = 5). (D) Dead/living staining analysis of 4T1 cells. (E) Percentages of dead and living 4T1 cells (*n* = 5). (F) The ROS generation evaluation inside cells using fluorescence imaging. (G) HIF‐1α immunofluorescence staining images of 4T1 cells after treatments. (H) Fluorescence intensity of HIF‐1α staining for 4T1 cells (*n* = 5). (I) Evaluation of Cleaved‐caspase‐3, GSDME‐N and GSDME‐FL expression levels for various treated 4T1 cells. (Uncropped original images of WB were shown in Figure S8, Supporting information). Mean ± SD are presented in data, two‐tailed unpaired t test, **p < 0.01, ***p < 0.001.

The cellular uptake of OSPN and OSPN_pro_ by 4T1 cancer cells was first evaluated. The red fluorescence signals of nanoparticles could be detected inside cells after incubation with OSPN and OSPN_pro_ (Figure 3A), indicating the effective intracellular nanoparticle internalization. The intensity of fluorescence signals of OSPN_pro_‐treated cells was consistent to that of OSPN‐treated cells (Figure S6, Supporting information), which suggested that OSPN and OSPN_pro_ had a similar cellular uptake efficacy. The in vitro therapeutic efficacy was then studied. OSPN and OSPN_pro_ did not obviously reduce the cell viabilities of 4T1 cells after incubation (Figure 3B). This suggested the negligible therapeutic efficacy for sole OSPN and OSPN_pro_. After nanoparticle incubation and US treatment, the cell viability was reduced to 51.1% for OSPN + US and 26.2% for OSPN_pro_ + US groups (Figure 3C). The therapeutic efficacy of SDT‐combined pyroptosis in OSPN_pro_ + US group was increased compared to sole SDT in OSPN + US group. The therapeutic efficacy was verified using dead/living staining of cells after treatments. Only green fluorescence stained living cells were found in PBS, OSPN, OSPN_pro_, and PBS + US groups, and dead cells (red fluorescence signals) were detected for OSPN + US and OSPN_pro_ + US groups (Figure 3D). The numbers of dead cells in OSPN_pro_ + US group were higher than those in OSPN + US group. The quantification analysis showed that the percentages of dead cells in OSPN_pro_ + US group was 78.6%, higher than that in OSPN + US (44.4%) group (Figure 3E). In the other four group, the percentages of dead cell were 0%. These results confirmed the better therapeutic efficacy of OSPN_pro_ over OSPN.

The intracellular ROS generation was then confirmed by fluorescence imaging. The ROS signals (green fluorescence) were only found in OSPN + US and OSPN_pro_ + US groups (Figure 3F). This verified the intracellular ROS generation after nanoparticle incubation and US treatment. The intensity of ROS staining signals in OSPN + US and OSPN_pro_ + US groups was almost consistent (Figure S7, Supporting information), which should be due to the similar cellular uptake efficacy and ^1^O_2_ generating efficacy for OSPN and OSPN_pro_. Stronger hypoxia‐inducible factor 1‐α (HIF‐1α) staining fluorescence signals were detected for PBS and PBS + US groups, but the HIF‐1α signals reduced for OSPN and OSPN_pro_ groups (Figure 3G), which suggested that OSPN and OSPN_pro_ could effectively alleviate hypoxia condition by carrying oxygen. The HIF‐1α staining fluorescence intensity was measured to be around 9.2 for both PBS and PBS + US group, while which was less than 0.9 for OSPN and OSPN_pro_ groups (Figure 3H). The expression levels of pyroptosis‐related biomarkers were then evaluated using western blotting (WB) analysis. Compared to other group, OSPN_pro_ plus US treatment could elevate the expressions of Cleaved‐caspase‐3 and GSDME‐N, while OSPN plus US treatment did not have obvious influences on their expression levels (Figure 3I and Figure S8, Supporting information). Moreover, the expression levels of GSDME‐FL in OSPN_pro_ + US group was reduced compared to that in PBS group. Thus, pyroptosis was triggered after OSPN_pro_ plus US treatment.

### 2.3 Deep‐tissue antitumor efficacy evaluation

**FIGURE 4:**
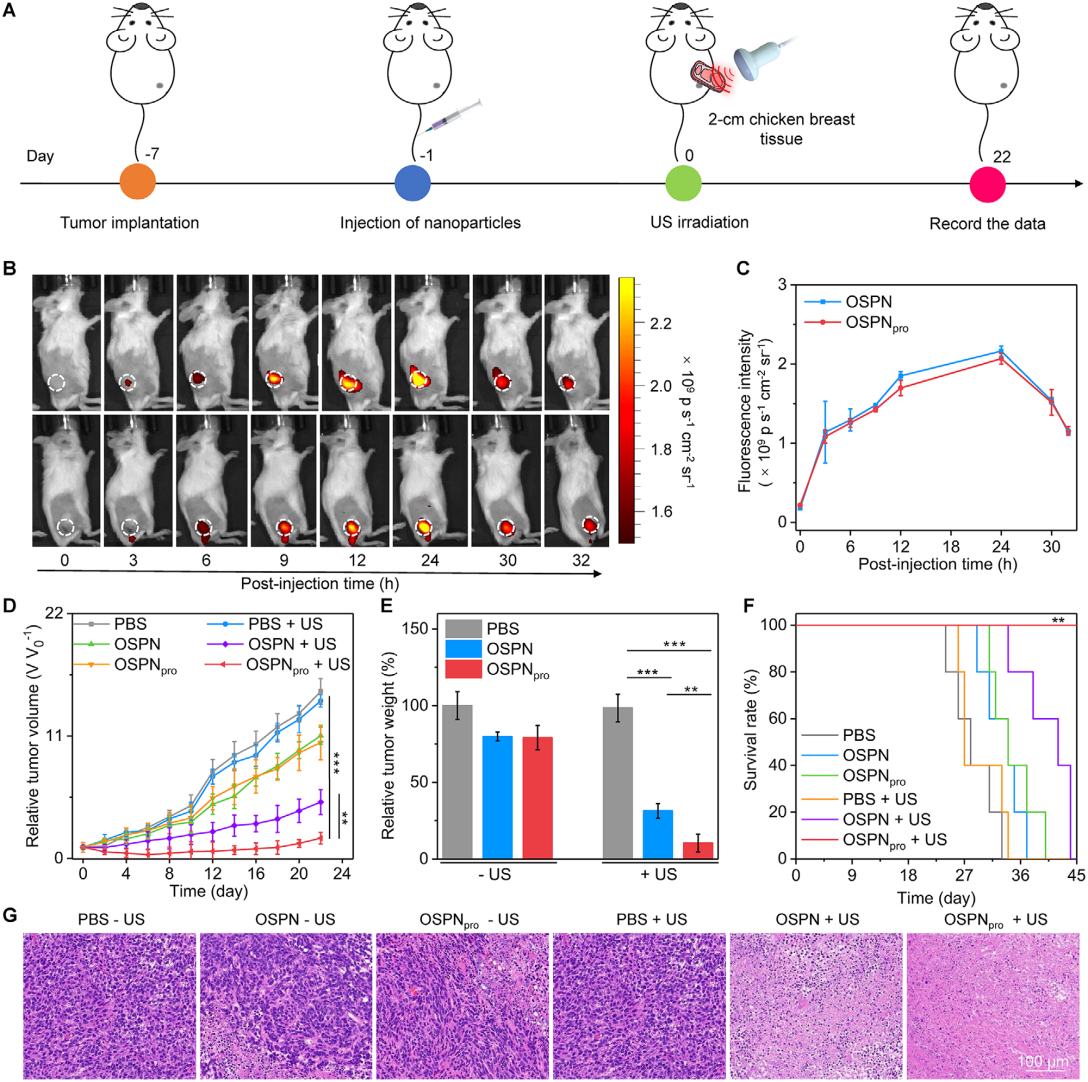
Deep‐tissue antitumor efficacy evaluation. (A) Schematic illustration of in vivo therapeutic efficacy evaluation using deep‐tissue tumors. (B) IVIS fluorescence imaging analysis of 4T1 tumor‐bearing mice with OSPN and OSPN_pro_ injection (white dotted circles indicated the tumor tissues). (C) Intensity of tumor fluorescence signals for mice with OSPN and OSPN_pro_ injection (*n* = 3). (D) Tumor volumes in various treated and control groups (*n* = 5). (E) Tumor weight data collected from various treated and control mice (*n* = 5). (F) Analysis of 4T1 tumor‐bearing mouse survival (*n* = 5). (G) Tumor H&E staining analysis after various treatments and without treatment. Mean ± SD are presented in data, two‐tailed unpaired *t* test, **p < 0.01, ***p < 0.001.

To evaluate deep‐tissue antitumor efficacy, 2‐cm chicken breast tissue covered subcutaneous 4T1 tumors were treated by US, followed by monitoring of tumor growth (Figure 4A). The accumulation of nanoparticles in tumor sites and bio‐distribution of nanoparticles after systematic injection through tail vein were studied. Because of the nanoparticle enrichments, fluorescence signals of tumor sites brightened after injection of OSPN and OSPN_pro_ (Figure 4B). Fluorescence intensity of tumor sites increased from around 0.2 × 10^9^ p s^−1^ cm^−2^ sr^−1^ (at 0 h) to 2.1 × 10^9^ and 2.0 × 10^9^ p s^−1^ cm^−2^ sr^−1^ (at 24 h) for OSPN‐ and OSPN_pro_‐injected mice, respectively (Figure 4C). The tumor accumulating efficacy of OSPN and OSPN_pro_ was consistent at all the same injection points. Their similar small sizes and abundant PEG surface coatings allowed such a high tumor accumulation efficiency for both OSPN and OSPN_pro_ by enhanced permeability and retention (EPR) effect. Ex vivo imaging data demonstrated that obvious signals could only be found in livers and tumors for OSPN‐ and OSPN_pro_‐injected mice after systematic injection (Figure S9, Supporting information). The fluorescence intensities of livers (around 3.3 × 10^9^ p s^−1^ cm^−2^ sr^−1^) and tumors (around 3.1 × 10^9^ p s^−1^ cm^−2^ sr^−1^) were much higher than those in spleens, hearts, lungs, and kidneys. Therefore, both OSPN and OSPN_pro_ displayed major bio‐distribution in tumors and livers.

The chicken breast tissue‐covered tumors of mice after systematic injection of nanoparticles were treated by US at 24 h and the tumor growths were monitored. OSPN + US and OSPN_pro_ + US treatment could remarkably inhibit the growths of tumors compared to the other groups, while sole OSPN and OSPN_pro_ injection failed to inhibit the tumor growths (Figure 4D). After 22 days of treatments, the tumor growths in OSPN_pro_ + US group was inhibited by 8.3‐fold, but which in OSPN + US group was inhibited by around 3.0‐fold. Obvious decrease in tumor weights was observed for OSPN + US and OSPN_pro_ + US groups (Figure 4E). The mean tumor weights in OSPN_pro_ + US group were 3.2‐fold lower relative to those in OSPN + US group. The tumor inhibition rate in OSPN_pro_ + US group reached 89.6%, while which in OSPN + US group was only 68.5% (Figure S10, Supporting information). The survival rate of tumor‐bearing mice in OSPN_pro_ + US group remained 100% after 45 days, while which was 0% in control and the other treatment groups (Figure 4F). Hematoxylin and eosin (H&E) staining data demonstrated that apoptosis of tumor cells was clearly observed for OSPN + US and OSPN_pro_ + US groups, but apoptosis was hardly detected in other groups (Figure 4G). More severe cell apoptosis was observed in OSPN_pro_ + US group compared to that in OSPN + US group. These results verified that the antitumor effect in OSPN_pro_ + US group was much higher compared to OSPN + US group.

### 2.4 Evaluation of anti‐metastasis efficacy

**FIGURE 5:**
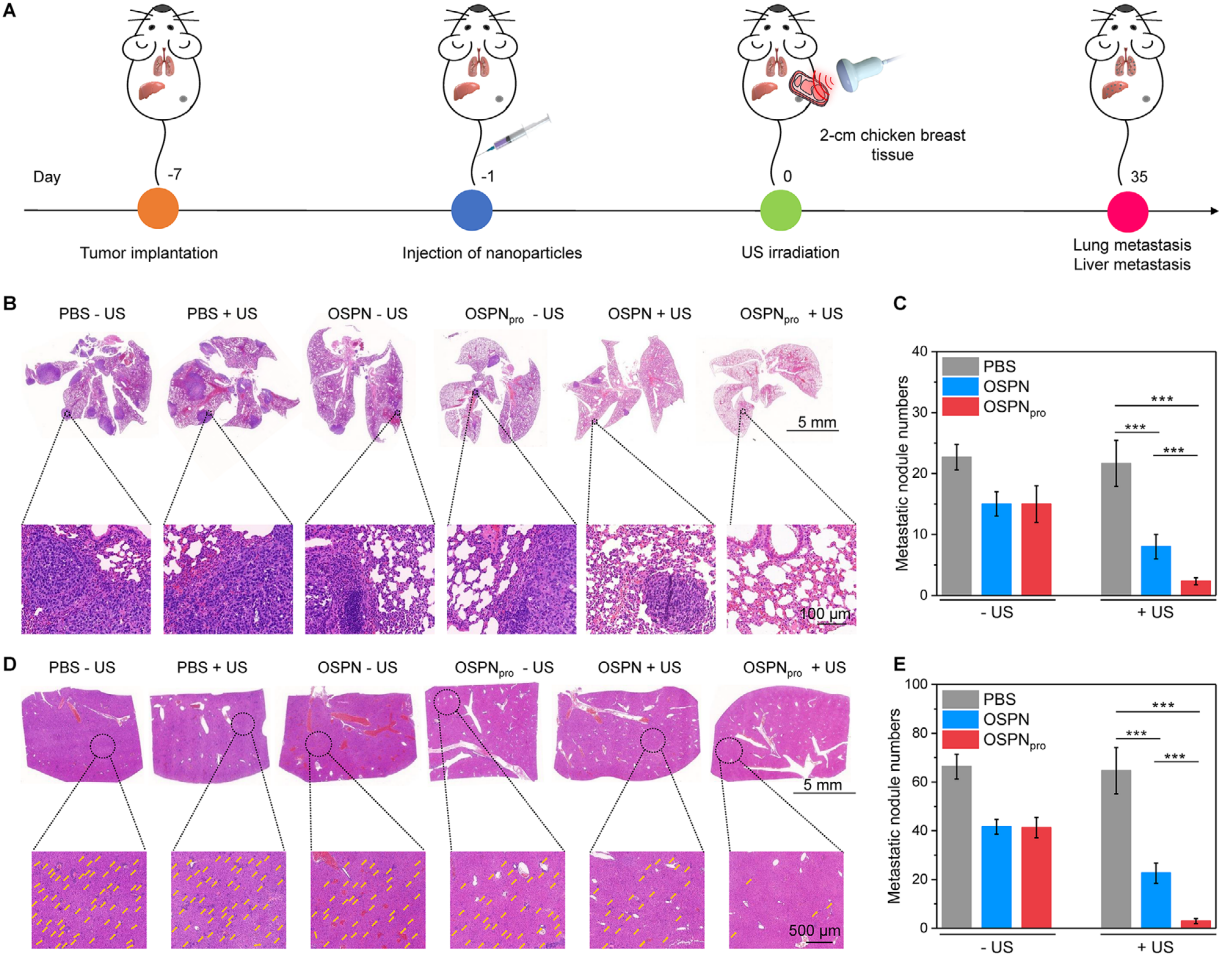
Evaluation of anti‐metastasis efficacy. (A) Schematic illustration of the evaluation of anti‐metastasis efficacy using deep‐tissue tumors. (B) H&E staining analysis of tumor metastasis in lungs. (C) Quantitative analysis of tumor metastasis in lungs (*n* = 5). (D) H&E staining analysis of tumor metastasis in livers. (E) Quantitative analysis of tumor metastasis in livers (*n* = 5). Mean ± SD are presented in data, two‐tailed unpaired *t* test, ***p < 0.001.

The anti‐metastasis efficacy was evaluated after various treatments of deep‐tissue tumors for 35 days (Figure 5A). Lungs in PBS, OSPN, OSPN_pro_, PBS + US, and OSPN + US groups showed the metastatic tumor nodules in varying degrees, while no obvious tumor metastasis was observed for OSPN_pro_ + US group (Figure 5B). Although the metastatic tumors in OSPN + US group were reduced compared to the control group, the number was still as high as 8.1 (Figure 5C). In contrast, the number for OSPN_pro_ + US group was 2.0. Histological staining analysis of livers suggested that the metastatic tumors were found in all groups, but tumor metastasis in OSPN_pro_ + US group was the weakest (Figure 5D). The number of tumors in OSPN_pro_ + US group was as low as 3.2, and 22.6 in OSPN + US group, while which was more than 41.3 for the other groups (Figure 5E).

In OSPN + US group, the tumor growths were obviously inhibited by sole SDT, and thus tumor metastasis in lung and liver was suppressed, but the anti‐metastasis efficacy was limited. In contrast, SDT‐combined pyroptosis was triggered in OSPN_pro_ + US group, which resulted in the activation of pyroptosis‐based immune response. The antitumor immunological effect thus not only improved the tumor inhibition efficacy, but also induced strong anti‐metastasis efficacy to remarkably suppress the tumor metastasis in lungs and livers.^[^ ^24^ ^]^ Therefore, OSPN_pro_‐mediated so‐pyroptosis possessed a higher anti‐metastasis efficacy than sole OSPN‐based SDT.

After monitoring for 22 days, all the mice were found to have a steady body weight (Figure S11, Supporting information). The other normal tissues (heart, kidney, and spleen) had healthy histological morphologies (Figure S12, Supporting information). OSPN_pro_‐mediated sono‐pyroptosis did not have obvious influence on mouse body weights and normal tissue histological morphologies.

### 2.5 Evaluation of pyroptosis‐based immune response

**FIGURE 6:**
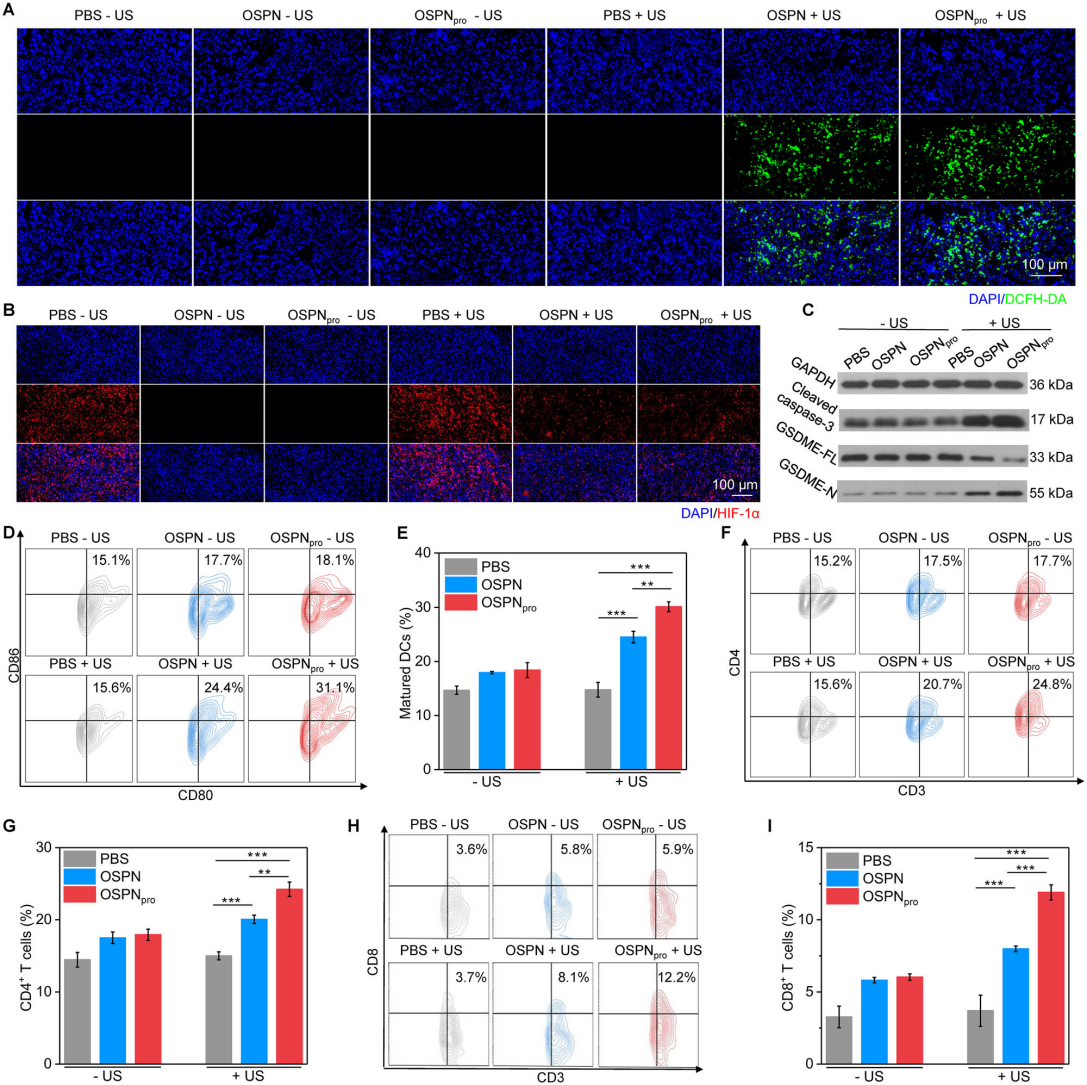
Evaluation of pyroptosis‐based immune response. (A) Evaluation of ^1^O_2_ generation inside tumors after various treatments using fluorescence imaging. (B) Evaluation of hypoxia conditions in tumors using immunofluorescence HIF‐1α staining. (C) Cleaved‐caspase‐3, GSDME‐N and GSDME‐FL expressions in tumors through WB analysis. (Uncropped original images of WB were shown in Figure S15, Supporting information). (D) Flow cytometry analysis of DCs. (E) Quantitative analysis of the percentages of DCs (*n* = 5). (F) Analysis of CD3^+^CD4^+^ T cells in tumor tissues using flow cytometry. (G) Quantitative analysis of the percentages of CD3^+^CD4^+^ T cells (*n* = 5). (H) Analysis of CD3^+^CD8^+^ T cells in tumor tissues using flow cytometry. (I) Quantitative analysis of the percentages of CD3^+^CD8^+^ T cells (*n* = 5). Mean ± SD are presented in data, two‐tailed unpaired t test, **p < 0.01, ***p < 0.001.

The generation of ^1^O_2_ in 2‐cm chicken breast tissue‐covered deep tumor tissues after various treatment was confirmed using fluorescence staining. As observed in the fluorescence images, green fluorescence signals of ^1^O_2_ were only found for OSPN + US and OSPN_pro_ + US (Figure 6A), indicating generation of ^1^O_2_ inside tumors after nanoparticle injection and US treatment. Tumors in other groups did not show any green fluorescence signals. Fluorescence intensities of ^1^O_2_ signals for OSPN + US and OSPN_pro_ + US were found to elevate by at least 5.8‐fold compared to other treatments (Figure S13, Supporting information). The hypoxic conditions in tumor sites after injection of PFH‐loaded nanoparticles were evaluated. Compared to control group, injection of OSPN and OSPN_pro_ remarkably reduced red fluorescence signals of HIF‐1α staining, which suggested the reduced expression levels of HIF‐1α inside tumor tissues (Figure 6B). Therefore, OSPN and OSPN_pro_ carrying oxygen could relieve the tumor hypoxia, which would promote SDT effect. The fluorescence signals of HIF‐1α staining for OSPN + US and OSPN_pro_ + US then elevated, which should be due to the fact that US‐mediated SDT further consumed oxygen. The fluorescence intensities of HIF‐1α staining in OSPN and OSPN_pro_ groups were around 16.7‐fold lower compared to control group (Figure S14, Supporting information).

To confirm the induction of pyroptosis, Cleaved‐caspase‐3, GSDME‐N and GSDME‐FL expressions in tumor tissues after OSPN_pro_ plus US treatment were investigated. Cleaved‐caspase‐3 and GSDME‐N expressions in OSPN_pro_ + US group were significantly upregulated compared to those in control group, while which in the other treatment groups were similar (Figure 6C and Figure S15, Supporting information). The expression level of GSDME‐FL in OSPN_pro_ + US group was found to reduce, but did not have remarkable change in OSPN_pro_ and OSPN + US groups. These results suggested that pyroptosis was induced after OSPN_pro_ plus US treatment.

To verify the activation of pyroptosis‐based immune response, the levels of immune cells in treated mice were measured. The injection of OSPN and OSPN_pro_ plus US treatment were found to significantly increase the levels of dendritic cells (DCs) in tumor draining lymph nodes, but sole OSPN and OSPN_pro_ injection without US treatment did not affect the DC maturation (Figure 6D). DC levels in OSPN_pro_ + US group was as high as 30.1%, higher than that in OSPN + US (24.5%), and control (14.6%) groups (Figure 6E). The intratumoral T cell levels were also measured. Obvious elevations in CD3^+^CD4^+^ T cell levels were found for OSPN + US and OSPN_pro_ + US groups in comparison to those for the control group (Figure 6F). Particularly, the percentage of CD3^+^CD4^+^ T cells after OSPN_pro_ plus US treatment was increased to 24.2%, while which in OSPN + US group was only 20.0% (Figure 6G). OSPN and OSPN_pro_ injection and US treatment were also found to increase the intratumoral levels of CD3^+^CD8^+^ T cells in comparison to sole OSPN and OSPN_pro_ injection groups (Figure 6H). OSPN_pro_ + US group had the highest levels of CD3^+^CD8^+^ T cells (11.9%) (Figure 6I). These results verified that pyroptosis‐based immune response was triggered for OSPN_pro_ + US group, which should be responsible for the excellent antitumor and anti‐metastasis efficacies.

## 3 CONCLUSION

We have reported the development of oxygen‐carrying semiconducting polymer nanoprodrugs (OSPN_pro_) to combine amplified SDT with pyroptosis for deep‐tissue cancer therapy. OSPN_pro_ were constructed to contain three major components: an oxygen carrier, a sonodynamic semiconducting polymer and ROS‐responsive DOX‐TK prodrug. The tumor hypoxia was relieved by OSPN_pro_ after their effective accumulation into tumors after systemic administration, which promoted the SDT effect of semiconducting polymer under US irradiation with generation of ^1^O_2_. The formed ^1^O_2_ activated ROS‐responsive DOX‐TK prodrugs to trigger pyroptosis that could induce the priming and activation of effector immune cells. In view of the good penetration depth of US, such OSPN_pro_‐based sono‐pyroptosis was used to treat 2‐cm chicken breast tissue‐covered deep tumors, leading to effective growth inhibition of tumors and suppression of the tumor metastasis. This work should present the first prodrug‐based organic nanoplatform for tumor intervention via combining SDT with controlled pyroptosis. OSPN_pro_ will be available for treatments of different types of deep‐seated tumors with enhanced antitumor and anti‐metastasis efficacies after combinations with immunotherapeutic drugs.

## AUTHOR CONTRIBUTIONS

J. Li, S. Peng, L. Sutrisno, and X. Lou conceived the idea, designed the experiments, and supervised the research. F. Wang, Y. Fan, and Y. Liu performed the experiments and analyzed the data. F. Wang wrote the draft, and J. Li and L. Sutrisno revised the manuscript.

## CONFLICT OF INTEREST STATEMENT

The authors declare no conflicts of interest.

## ETHICS STATEMENT

All animal experiments were conducted with the permissions of the Institutional Anima Care and Treatment Committee of Donghua University (approval number DHUEC‐NSFC‐2022‐16).

## Supporting information

Supporting Information

## Data Availability

All data related to this study are present in the article. Any other data associated with this work are available from the corresponding authors upon request.
