# Erratum to “Oxygen‐carrying semiconducting polymer nanoprodrugs induce sono‐pyroptosis for deep‐tissue tumour treatment”

**DOI:** 10.1002/EXP.20240339

**Published:** 2024-10-28

Wang F., Fan Y., Liu Y., et al. Oxygen‐carrying semiconducting polymer nanoprodrugs induce sono‐pyroptosis for deep‐tissue tumour treatment, *Exploration*, 2024, *4*, 20230100.

In our manuscript titled “Oxygen‐carrying semiconducting polymer nanoprodrugs induce sono‐pyroptosis for deep‐tissue tumour treatment”, H&E staining image of PBS + US group in Figure 4G was misplaced due to an inadvertent error. The error was spotted in time after revisiting the article and we correct this error by providing the revised image.

**FIGURE 4:**
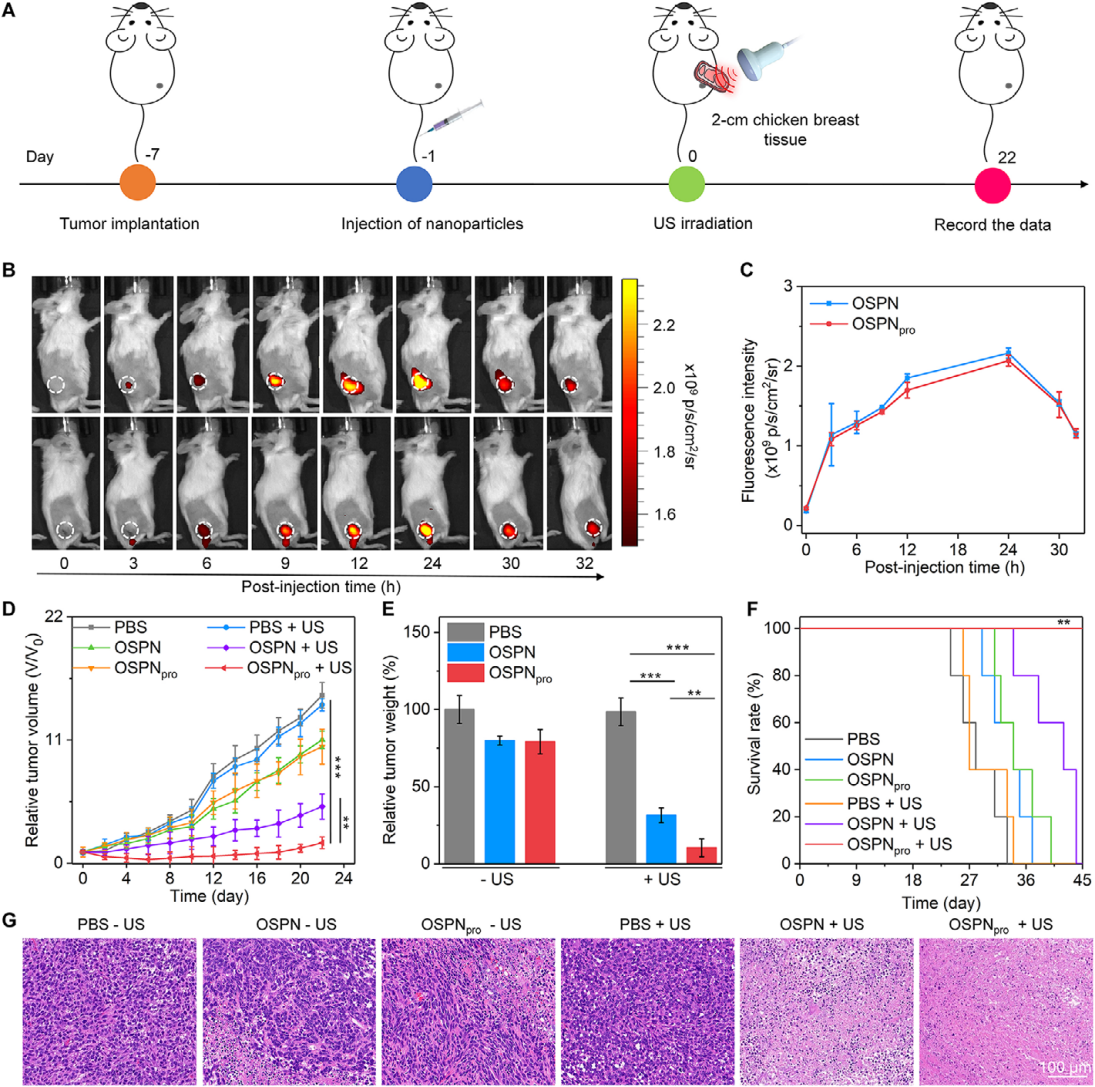
Deep‐tissue antitumour efficacy evaluation. (A) Schematic illustration of in vivo therapeutic efficacy evaluation using deep‐tissue tumours. (B) IVIS fluorescence imaging analysis of 4T1 tumour‐bearing mice with OSPN and OSPN_pro_ injection (white dotted circles indicated the tumour tissues). (C) Intensity of tumour fluorescence signals for mice with OSPN and OSPN_pro_ injection (*n* = 3). (D) Tumour volumes in various treated and control groups (*n* = 5). (E) Tumor weight data collected from various treated and control mice (*n* = 5). (F) Analysis of 4T1 tumour‐bearing mouse survival (*n* = 5). (G) Tumour H&E staining analysis after various treatments and without treatment. Mean ± SD are presented in data, two‐tailed unpaired *t* test, **p < 0.01, ***p < 0.001.

The revised Figure 4

We apologize for this error.

