# Supplementary material for: Oxygen‐carrying semiconducting polymer nanoprodrugs induce sono‐pyroptosis for deep‐tissue tumor treatment
Source: Exploration (Beijing). 2024 Feb 19;4(4):20230100. doi: 10.1002/EXP.20230100 (PMC11335461; doi:10.1002/EXP.20230100)
Supplement: Supplementary file 1 — Supporting Information [file EXP2-4-20230100-s001.docx]

**Supporting information**

**Oxygen-carrying semiconducting polymer** **nanoprodrugs induce sono-pyroptosis for deep-tissue tumor treatment**

Fengshuo Wang^1^, Yongliang Fan^2^, Yue Liu^1^, Xiangxin Lou^1^*, Linawati Sutrisno^3^*, Shaojun Peng^4^*, Jingchao Li^1^*

^1^ State Key Laboratory for Modification of Chemical Fibers and Polymer Materials, College of Biological Science and Medical Engineering, Donghua University, Shanghai 201620, China

^2^ Department of Cardiovascular Surgery, Shanghai General Hospital, Shanghai Jiao Tong University School of Medicine, Shanghai 201600, China

^3^ World Premier International (WPI) Research Center for Materials Nanoarchitectonics (MANA), National Institute for Materials Science (NIMS), 1-1 Namiki, Tsukuba, 305-0044, Japan

^4^ Zhuhai Institute of Translational Medicine, Zhuhai Precision Medical Center, Zhuhai People’s Hospital (Zhuhai hospital affiliated with Jinan University), Zhuhai, Guangdong 519000, China

Fengshuo Wang and Yongliang Fan contributed equally to this work.

*Corresponding authors:

xiangxin@dhu.edu.cn (X. Lou), SUTRISNO.Linawati@nims.go.jp (L. Sutrisno), henry2008_ok@126.com (S. Peng), jcli@dhu.edu.cn (J. Li).

**EXPERIMENTAL SECTION**

**Materials**

SOSG was purchased from Molecular Probes Lnc. (USA). PFODBT, DOX, PFH, PEG-b-PPG-b-PEG and 2’,7’-dichlorodihydrofluorescein diacetate (H_2_DCFDA) were purchased from Sigma Aldrich (USA). Antibodies were purchased from Abcam (USA) or Biolegend (USA).

**Characterization**

The prodrugs were characterized using^1^H NMR spectrometer. Measurements of sizes and zeta potentials were performed using a Nanoanalyzer (Malvern Zetasizer). TEM (Tecnai G2) was used to capture images. A TU-1810 Persee spectrophotometer was used to measure the UV-Vis absorbance. A SHIMADZU ﬂuorescence spectrophotometer was used to obtain fluorescence spectra. A LC-16 SHIMADZU high-performance liquid chromatography (HPLC) was used to analyze drug release.

**Synthesis of ROS-responsive prodrugs**

ROS-responsive TK linker (COOH-TK-COOH, 22.4 mg), 1-ethyl-3-(3-dimethylaminopropyl) carbodiimide (19.2 mg) and N-hydroxysuccinimide (11.6 mg) were dissolved in 5.0 mL tetrahydrofuran, and the resulting solution was stirred at room temperature for 3 h. DOX (60.0 mg) dissolved in 1.0 mL tetrahydrofuran was then added into above mixture and the obtained solution was stirred at room temperature for another 24 h. After purification and lyophilization, the ROS-responsive prodrugs (DOX-TK) were obtained.

**Synthesis of nanoprodrugs**

PEG-b-PPG-b-PEG (25.0 mg), PFODBT (0.5 mg), DOX-TK (0.1 mg), and PFH (0.5 mg) were dissolved in 1.0 mL tetrahydrofuran, and the solution was rapidly injected into a mixed solution of water/tetrahydrofuran (9.0 mL water and 1.0 mL tetrahydrofuran). After sonication, tetrahydrofuran was removed and nanoparticles were formed. The products were further purified by ultrafiltration to obtain OSPN_pro_. To synthesize control nanoparticles (OSPN) without loading of DOX-TK prodrugs, PFODBT (0.5 mg), PFH (0.5 mg) and PEG-b-PPG-b-PEG (25.0 mg) dissolved in tetrahydrofuran was injected into the water/tetrahydrofuran solution.

**Hemolysis assay**

Hemolysis was evaluated using mouse red blood cells after incubation with PBS (negative control), water (positive control), OSPN_pro_ or OSPN at various concentrations for 2 h.

**Sonodynamic effect evaluation**

OSPN_pro_ or OSPN solutions containing SOSG were treated by US (1.0 MHz, 1.0 W cm^-2^, 50% duty) for 1, 2, 3, 4, 5, 6, 7, 8, 9 and 10 min. The fluorescence spectra of these solutions without or with US treatment were recorded. The fluorescence intensities were used to verify the ^1^O_2_ generation of sonodynamic effect.

**Evaluation of prodrug activation**

OSPN_pro_ solution was treated by US (1.0 MHz, 1.0 W cm^-2^, 50% duty) for 5, 10 and 15 min, and the release of active DOX was measured using HPLC.

**Cell viability and therapeutic efficacy evaluation**

4T1 cancer cells were incubated with OSPN or OSPN_pro_ at various concentrations for 24 h, and the cell viability of 4T1 cells was measured via CCK-8 analysis. To evaluate in vitro therapeutic efficacy, 4T1 cells after incubation with OSPN or OSPN_pro_ were treated by US (1.0 MHz, 1.0 W cm^-2^, 50% duty) for 5 min, and then the cell viability was measured.

**Intracellular ROS level evaluation**

4T1 cancer cells after incubation with OPSN or OSPN_pro_ (25 μg mL^-1^) for 24 h were further incubated with H_2_DCFDA. Then the cells were treated by US (1.0 MHz, 1.0 W cm^-2^, 50% duty) for 5 min, the intracellular ROS levels were evaluated via observing the green fluorescence signals of cells.

**Evaluation of hypoxia condition at the cellular level**

4T1 cells were co-incubated with OSPN or OSPNpro (25 μg mL^-1^) for 12 h in the oxygen-deprived chambers, then treated with US (1.0 MHz, 1.0 W cm^-2^, 50% duty) for 5 min. The cells were used for HIF-1α immunofluorescence staining.

**Evaluation of pyroptosis at the cellular level**

4T1 cancer cells after incubation with OSPN or OSPN_pro_ (25 μg mL^-1^) for 24 h were treated by US (1.0 MHz, 1.0 W cm^-2^, 50% duty) for 5 min. Pyroptosis was evaluated by measuring the expression levels of Cleaved-caspase-3, GSDME-FL and GSDME-N via WB assay.

**Tumor mouse model establishment**

The animal experiments were conducted with the permissions of the Institutional Anima Care and Treatment Committee of Donghua University (approval number DHUEC-NSFC-2022-16)*.* 4T1 cells were subcutaneously injected into right flank of BALB/c mouse (female, 4-6 weeks) to establish tumor mouse models.

**In vivo tumor accumulation and biodistribution evaluation**

After intravenous injection with OSPN and OSPN_pro_ (300 μg mL^-1^), 4T1 tumor-bearing mice were imaged at designed timepoints. To study biodistribution, the mice receiving injection of OSPN and OSPN_pro_ were euthanized to extract kidney, liver, lung, spleen, heart, and tumors. These tissues were then imaged using the fluorescence imaging system.

**In vivo tumor inhibition efficacy evaluation**

4T1 tumor-bearing BALB/c mice were randomly divided into six groups, including: (1) PBS, (2) OSPN, (3) OSPN_pro_, (4) PBS  +  US, (5) OSPN  +  US, and (6) OSPN_pro_  +  US. PBS, OSPN or OSPN_pro_ (300 μg mL^-1^) were injected into mice via tail vein and the tumors were treated by US (1.0 MHz, 1.0 W cm^-2^, 50% duty) for 10 min. The lengths and widths of tumors were measured for 22 days to calculate the tumor volumes. At the endpoint of treatments, the mice were euthanized to extract tumors, and then photographs of tumors were taken and weights of tumors were recorded. The collected tumors were then used for histology analysis using H&E staining. The body weights of mice were recorded for 22 days after different treatments. H&E staining was conducted to evaluate the in vivo biosafety.

**In vivo anti-metastasis effect evaluation**

The 4T1 tumor-bearing BALB/c mice in 6 groups were injected with PBS, OSPN or OSPN_pro_ (300 μg mL^-1^), followed by treatment of tumors with US (1.0 MHz, 1.0 W cm^-2^, 50% duty) for 10 min. After treatments for 35 days, the mice were euthanized to extract lungs and livers. H&E staining of lungs and livers was conducted to evaluate anti-metastasis effect.

**Evaluation of tumor** **hypoxia condition**

The 4T1 tumor-bearing BALB/c mice in 6 groups were injected with PBS, OSPN or OSPN_pro_ (300 μg mL^-1^), followed by treatment of tumors with US (1.0 MHz, 1.0 W cm^-2^, 50% duty) for 10 min. The mice were euthanized to extract tumors after various treatments for 12 h, and the tumors were used for immunofluorescence staining of HIF-1α.

**Intratumoral ROS level evaluation**

After injection of PBS, OSPN or OSPN_pro_ (300 μg mL^-1^) into 4T1 tumor-bearing BALB/c mice via tail vein, SOSG was intratumorally injected into tumor sites, and then the tumors were treated by US (1.0 MHz, 1.0 W cm^-2^, 50% duty) for 10 min. Then the mice were euthanized to extract tumors and the tumors were cut into sections for cell nucleus staining by 4’,6-diamidino-2-phenylindole (DAPI). The intratumoral ROS levels were evaluated by observing the green fluorescence signals.

**Evaluation of pyroptosis levels in tumors**

After various treatments, 4T1 tumor-bearing BALB/c mice were euthanized to extract tumors. The tumors were used for WB assay to evaluate the expression levels of Cleaved-caspase-3, GSDME-FL and GSDME-N.

**Evaluation of immune response**

After various treatments, 4T1 tumor-bearing BALB/c mice were euthanized to extract tumors. The tumors were used to prepare single cell suspensions via enzyme digestion and filtration, followed by separation of lymphocytes using lymphocyte separation medium. The obtained single cells were stained with antibodies and then analyzed using a BD Biosciences flow cytometer.

**Statistical analysis**

The significant differences were calculated using a two-tailed unpaired t-test (GraphPad Prism 8.0), which were indicated as follows: * (p < 0.05), ** (p < 0.01), and *** (p < 0.001).

**
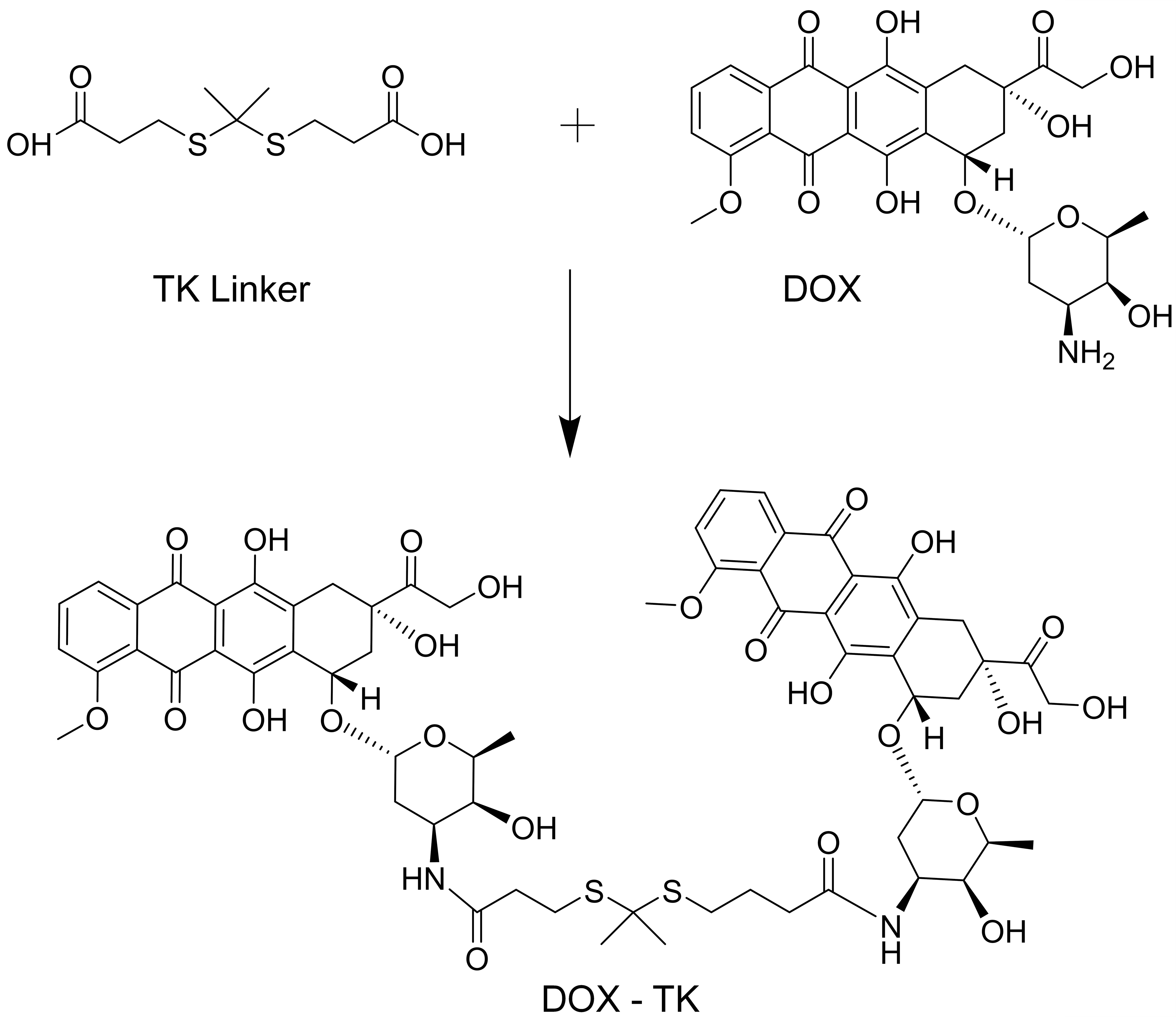
**

**Figure S1.** Synthetic route of DOX-TK.


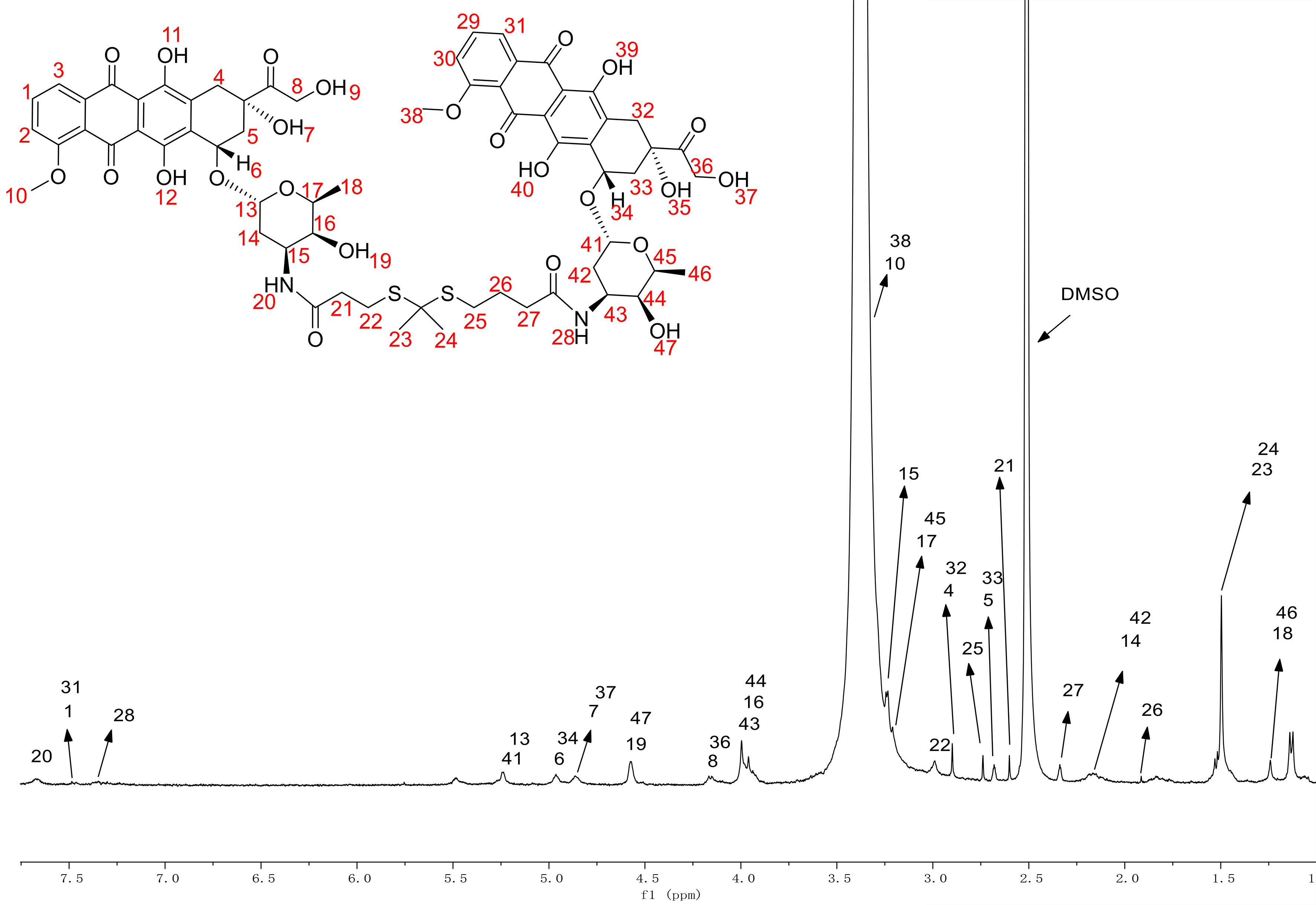


**Figure S2.** ^1^H NMR spectrum of DOX-TK in DMSO.


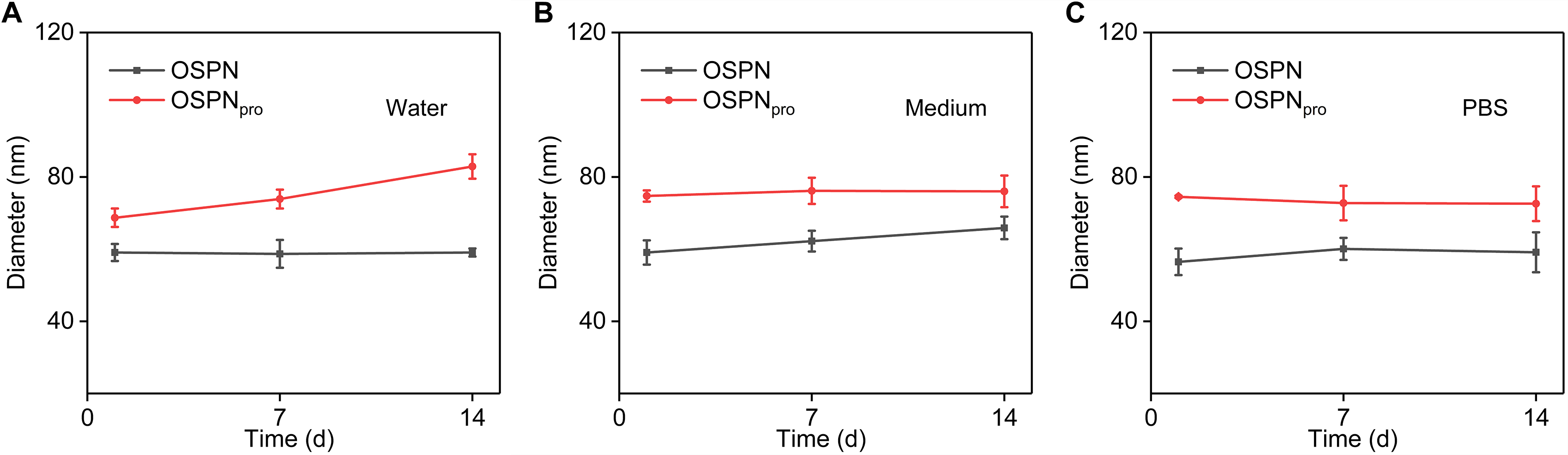


**Figure S3.** Colloidal stability evaluation by measuring the diameters of OSPN and OSPN_pro_ in water (A), cell culture medium (B) and PBS (C) for 14 days (n = 3). Mean ± SD are presented in data.


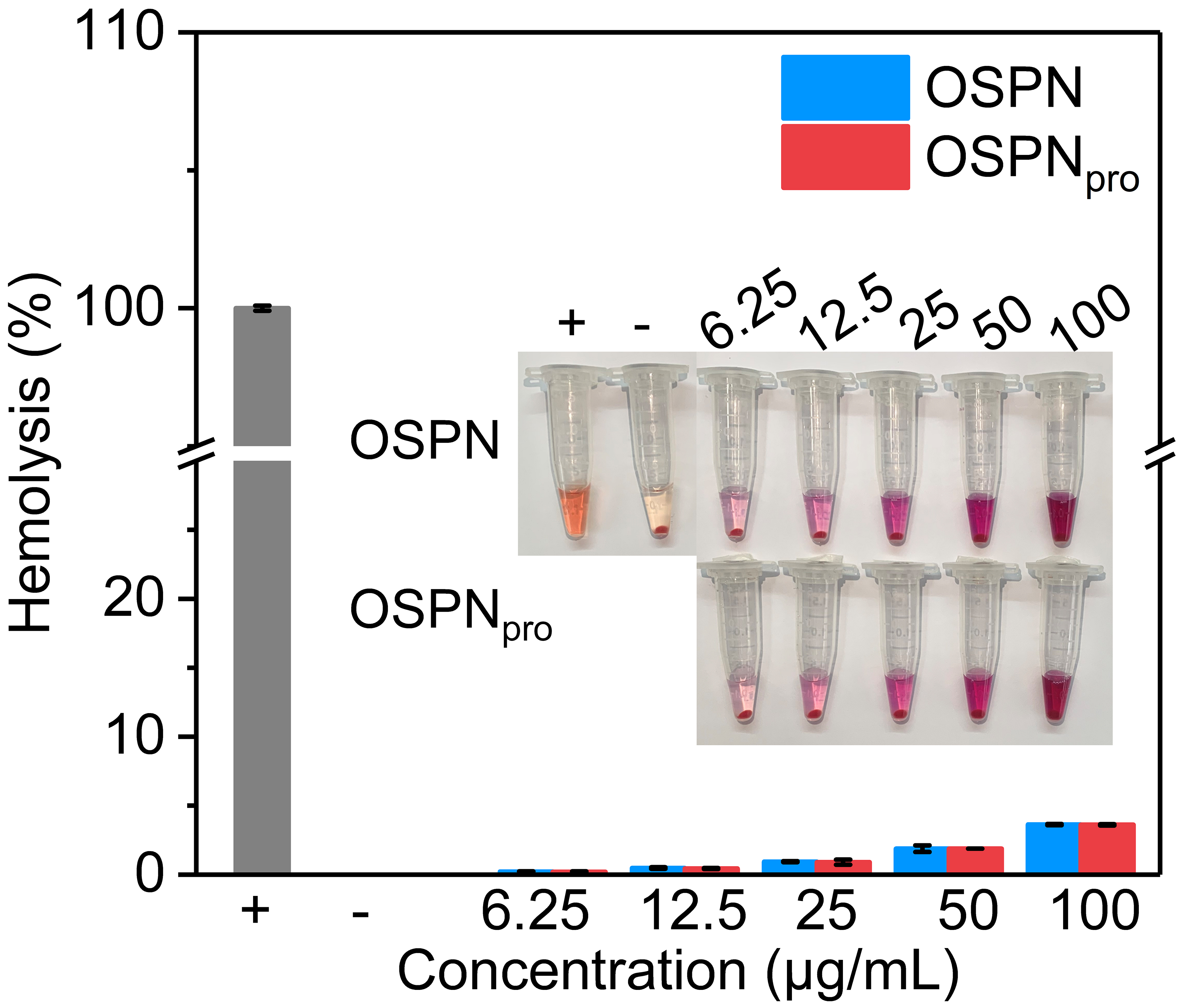


**Figure S4.** Hemolysis analysis of OSPN and OSPN_pro_ after incubation with red blood cells (n = 3). Mean ± SD are presented in data.


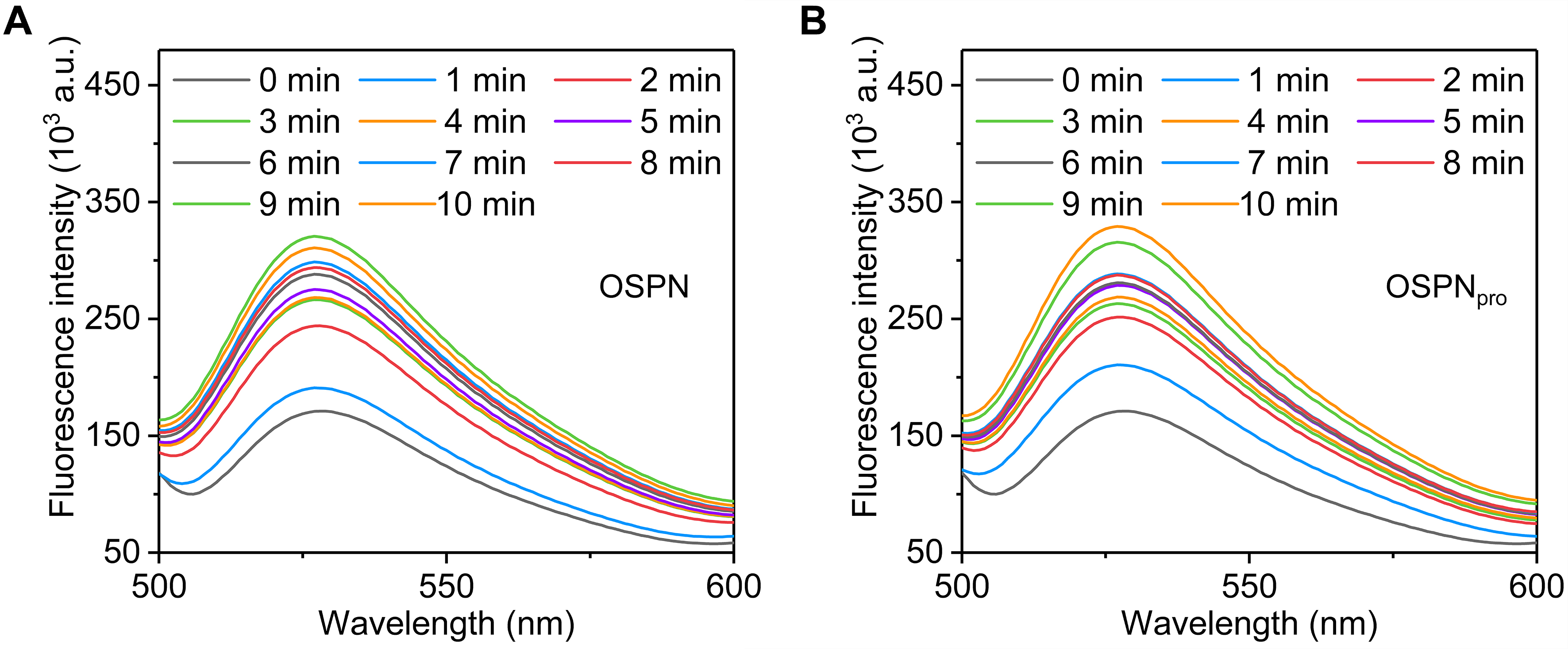


**Figure S5.** (A) Fluorescence spectra of SOSG in solution containing OSPN after US treatment for different time (excitation = 488 nm). (B) Fluorescence spectra of SOSG in solution containing OSPN_pro_ after US treatment for different time (excitation = 488 nm).


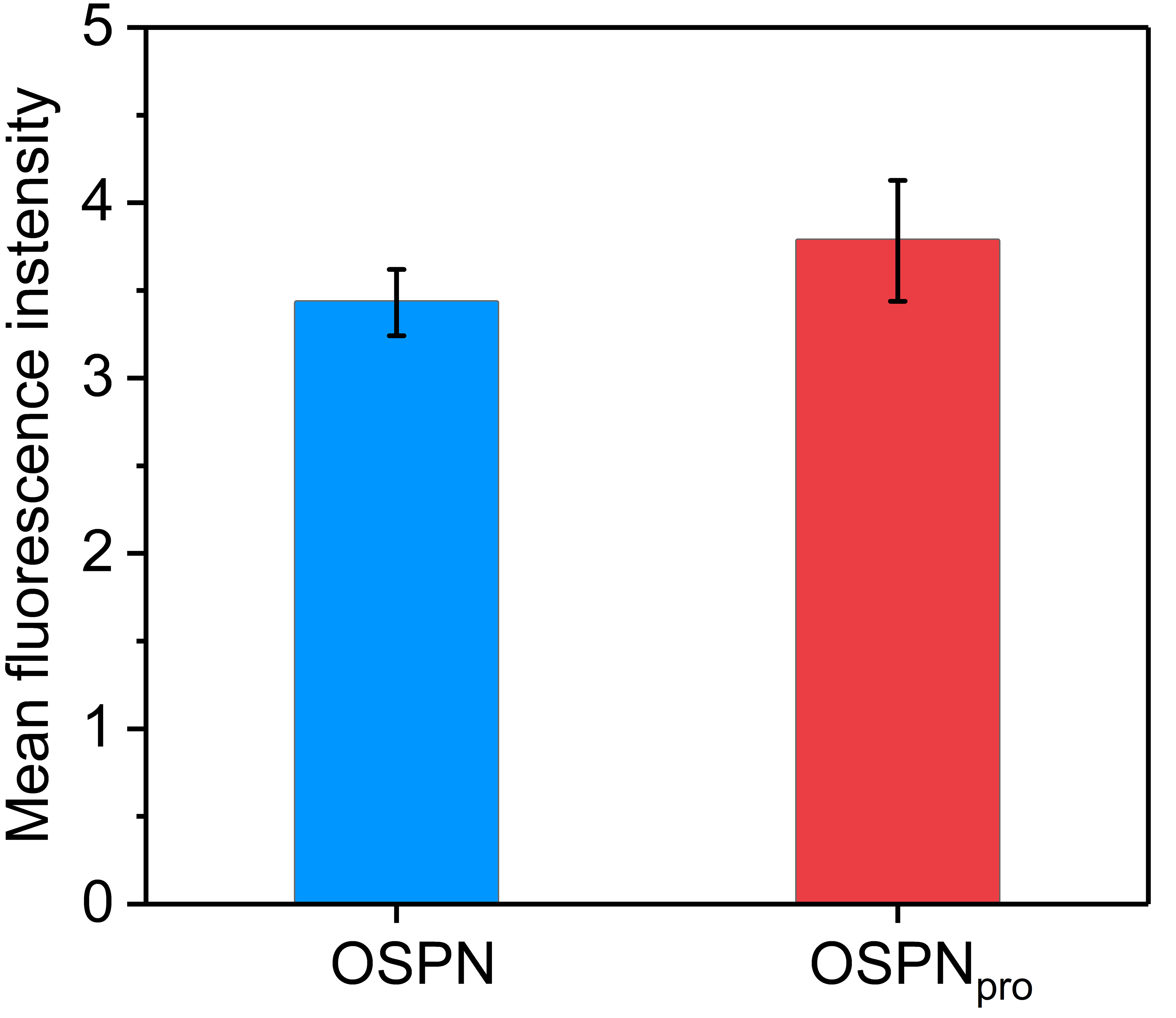


**Figure S6.** Relative fluorescence intensity of 4T1 cancer cells after different treatments (n = 5). Mean ± SD are presented in data.


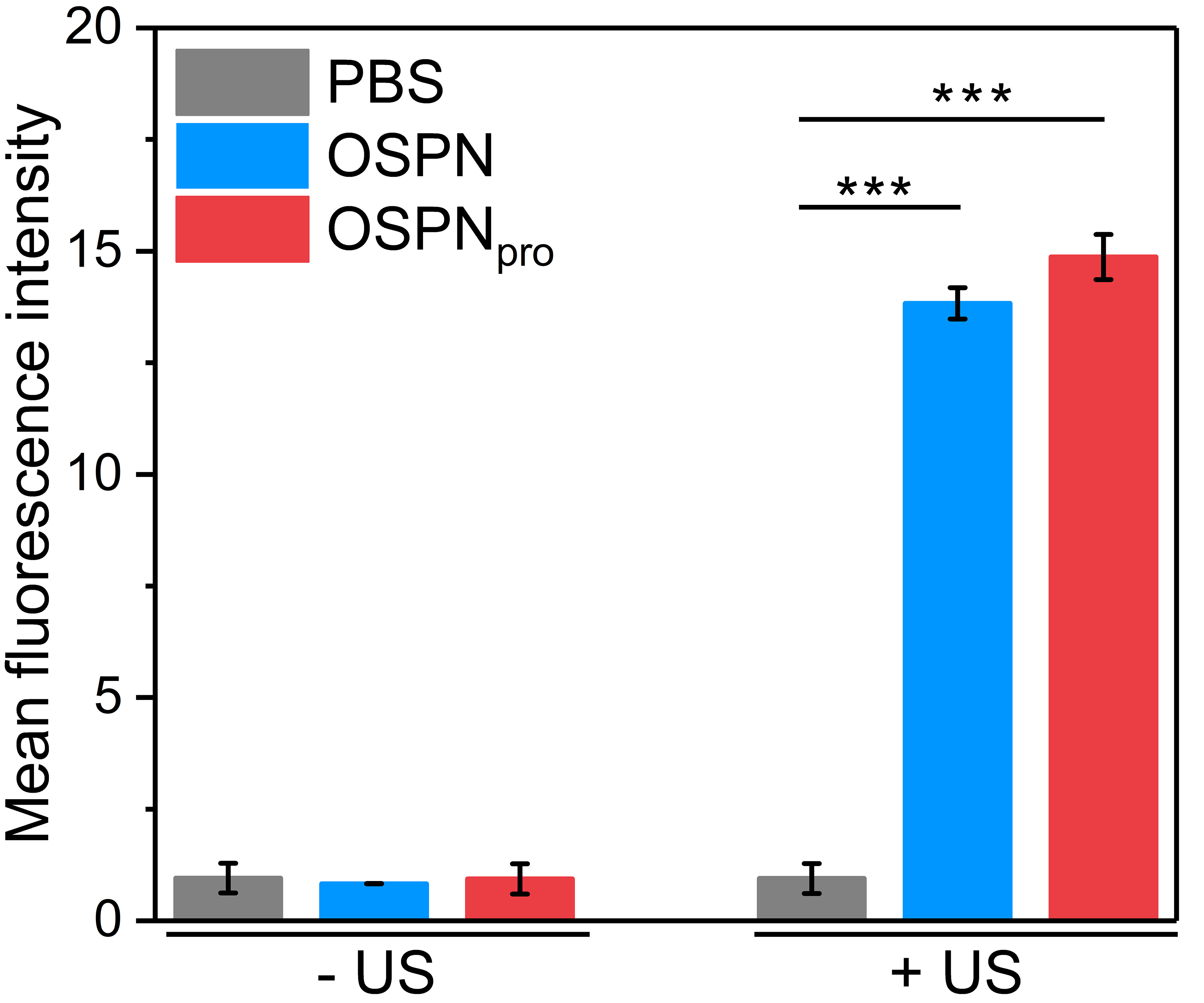


**Figure S7.** Relative fluorescence intensity of 4T1 cells after incubation with ROS probe (H_2_DCFDA) and different treatments (n = 5). Mean ± SD are presented in data, two-tailed unpaired t test, *** p < 0.001.

**Figure S8.** Uncropped original images of Cleaved-caspase-3, GSDME-FL and GSDME-N for 4T1 cells via WB analysis.


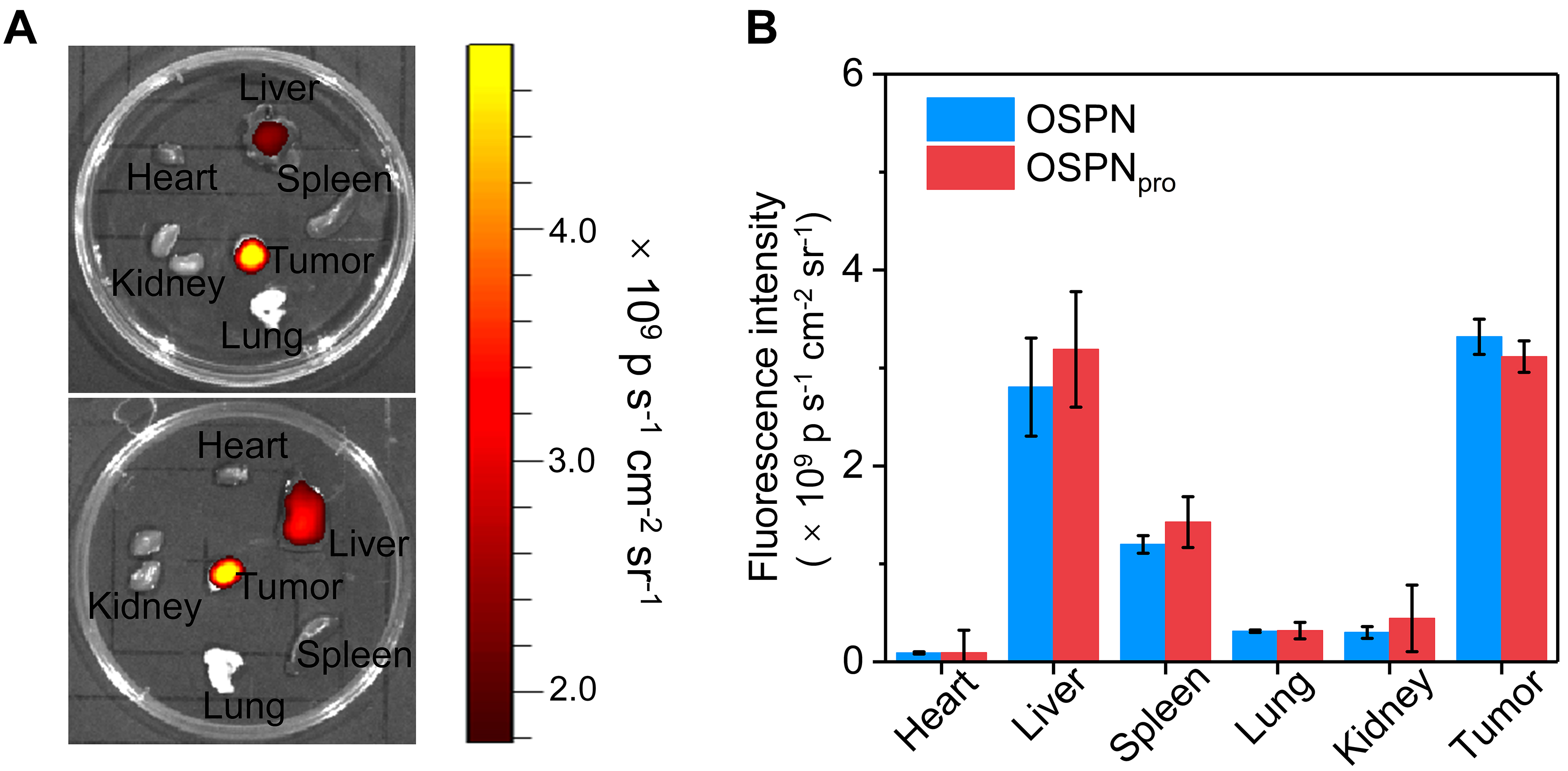


**Figure S9.** (A) Fluorescence images of heart, liver, spleen, lung, kidney and tumor after intravenous injection of OSPN and OSPN_pro_. (B) Fluorescence intensity of heart, liver, spleen, lung, kidney and tumor in different mice (n = 3). Mean ± SD are presented in data.


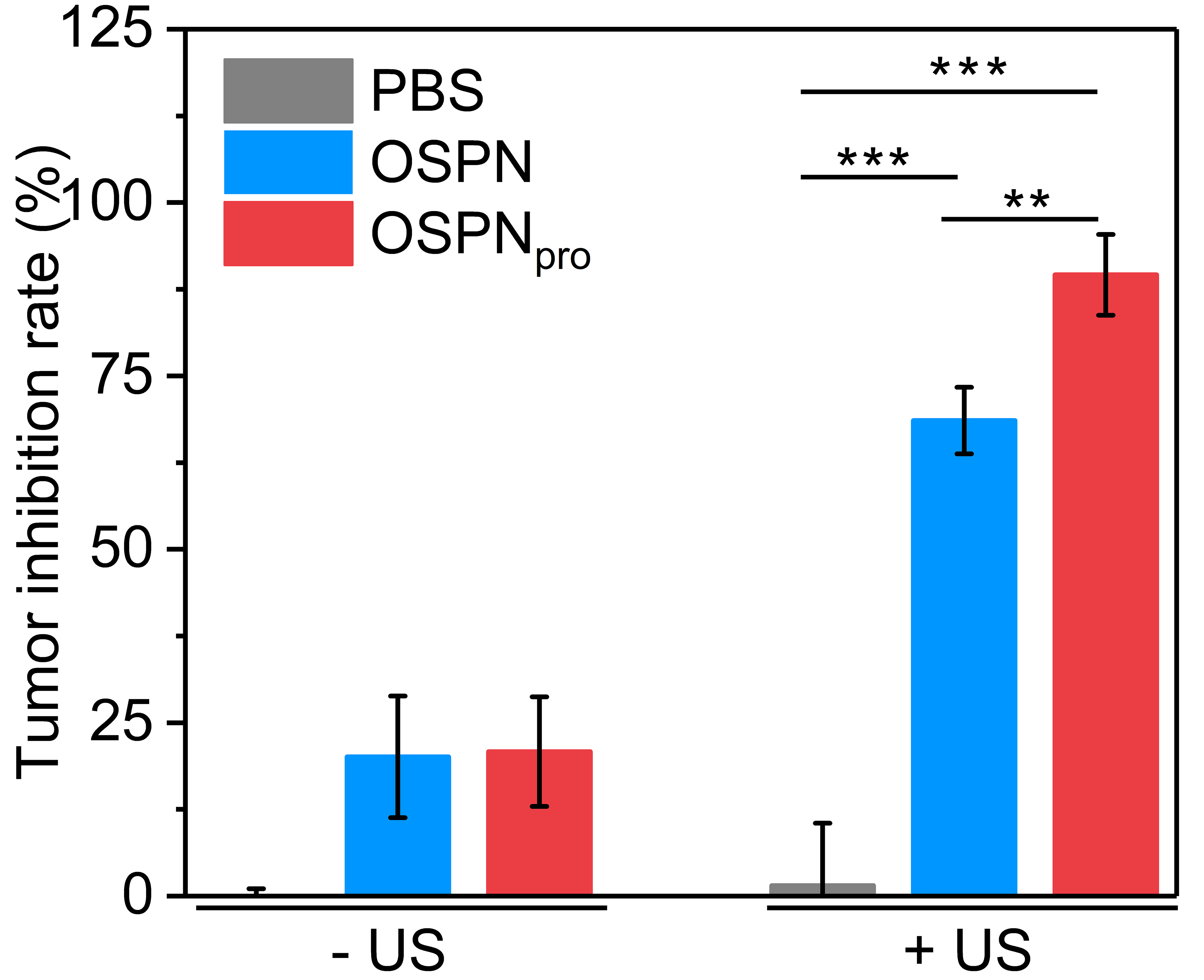


**Figure S10.** The tumor inhibition rate in various treated and control groups (n = 5). Mean ± SD are presented in data, two-tailed unpaired t test, ** p < 0.01, *** p < 0.001.


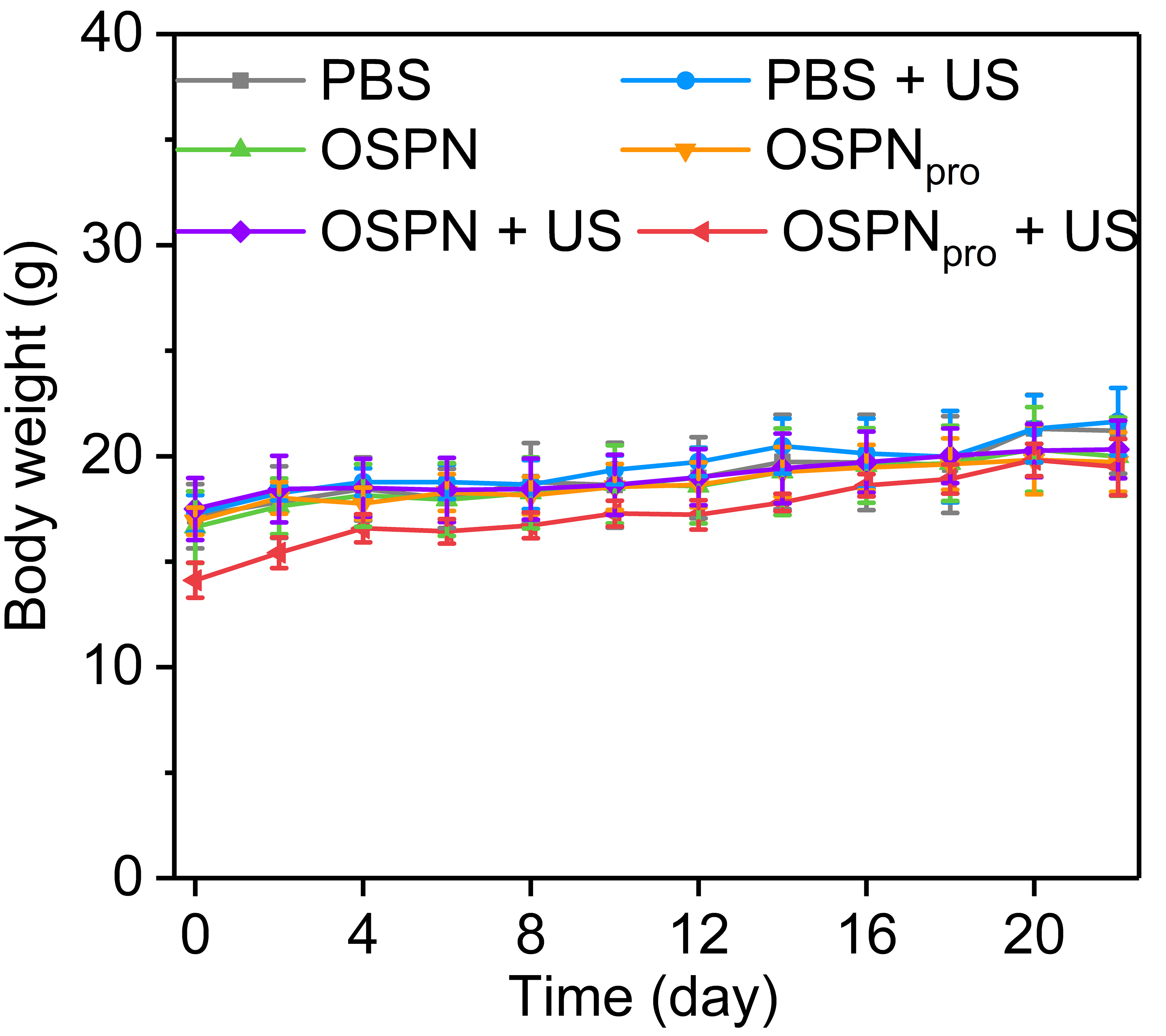


**Figure S11.** Body weight of 4T1 tumor-bearing BALB/c mice after intravenous injection of OSPN and OSPN_pro_ with or without US irradiation (n = 5). Mean ± SD are presented in data.


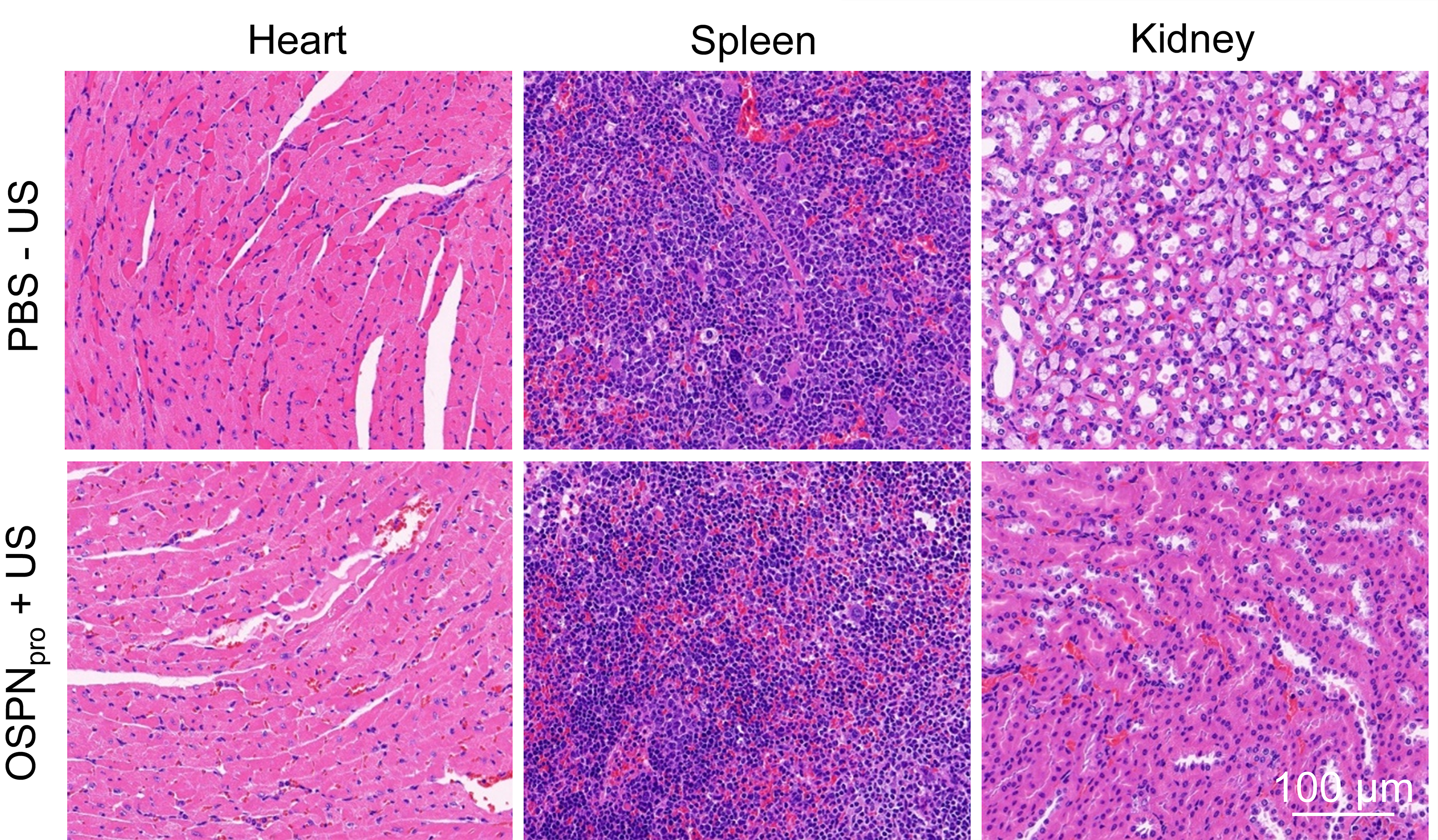


**Figure S12.** H&E staining images of heart, spleen and kidney of 4T1 tumor-bearing BALB/c mice in PBS and OSPN_pro_ + US groups.


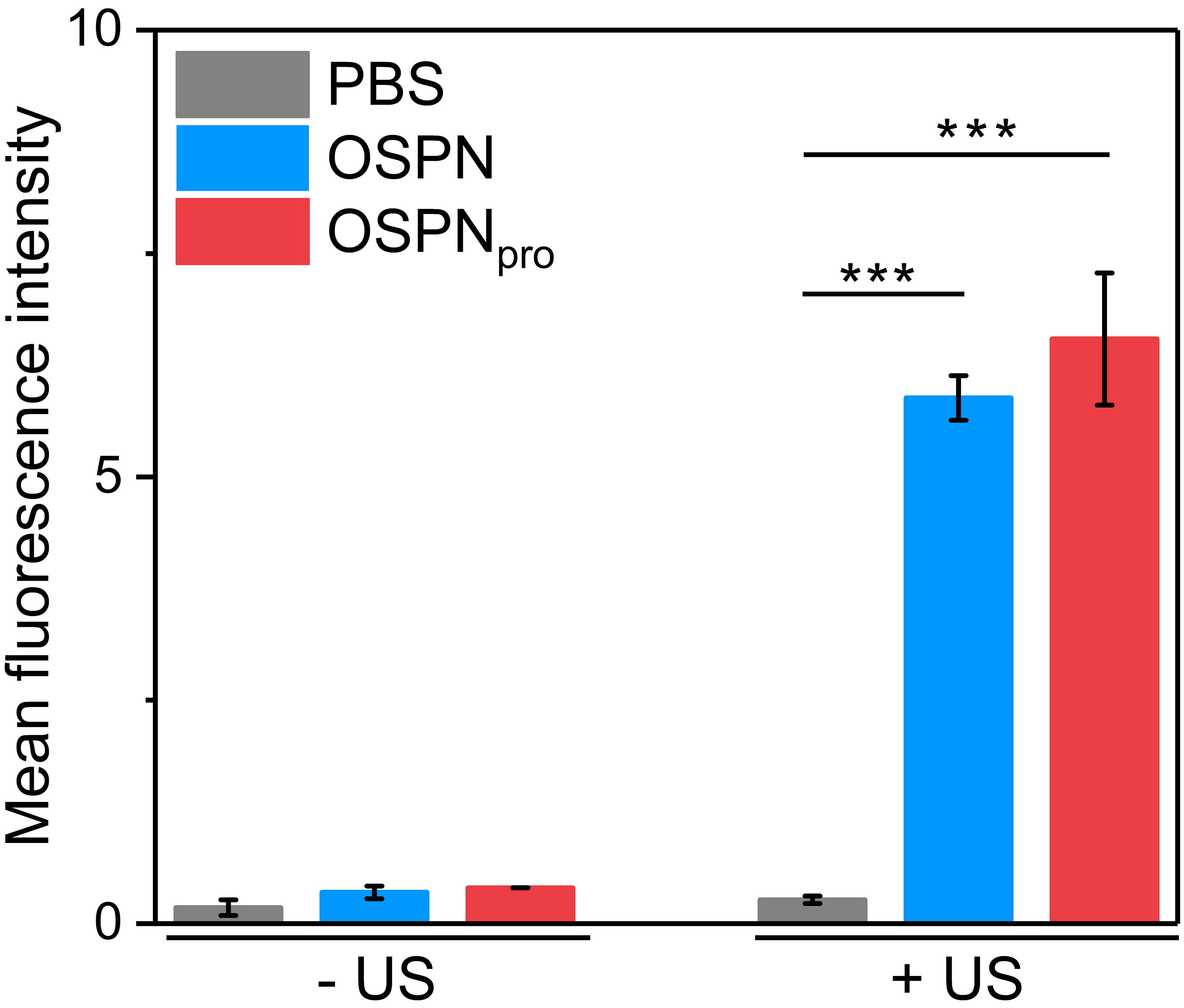


**Figure S13.** Mean fluorescence intensity of the produced ROS in tumors (n = 5). Mean ± SD are presented in data, two-tailed unpaired t test, *** p < 0.001.

**
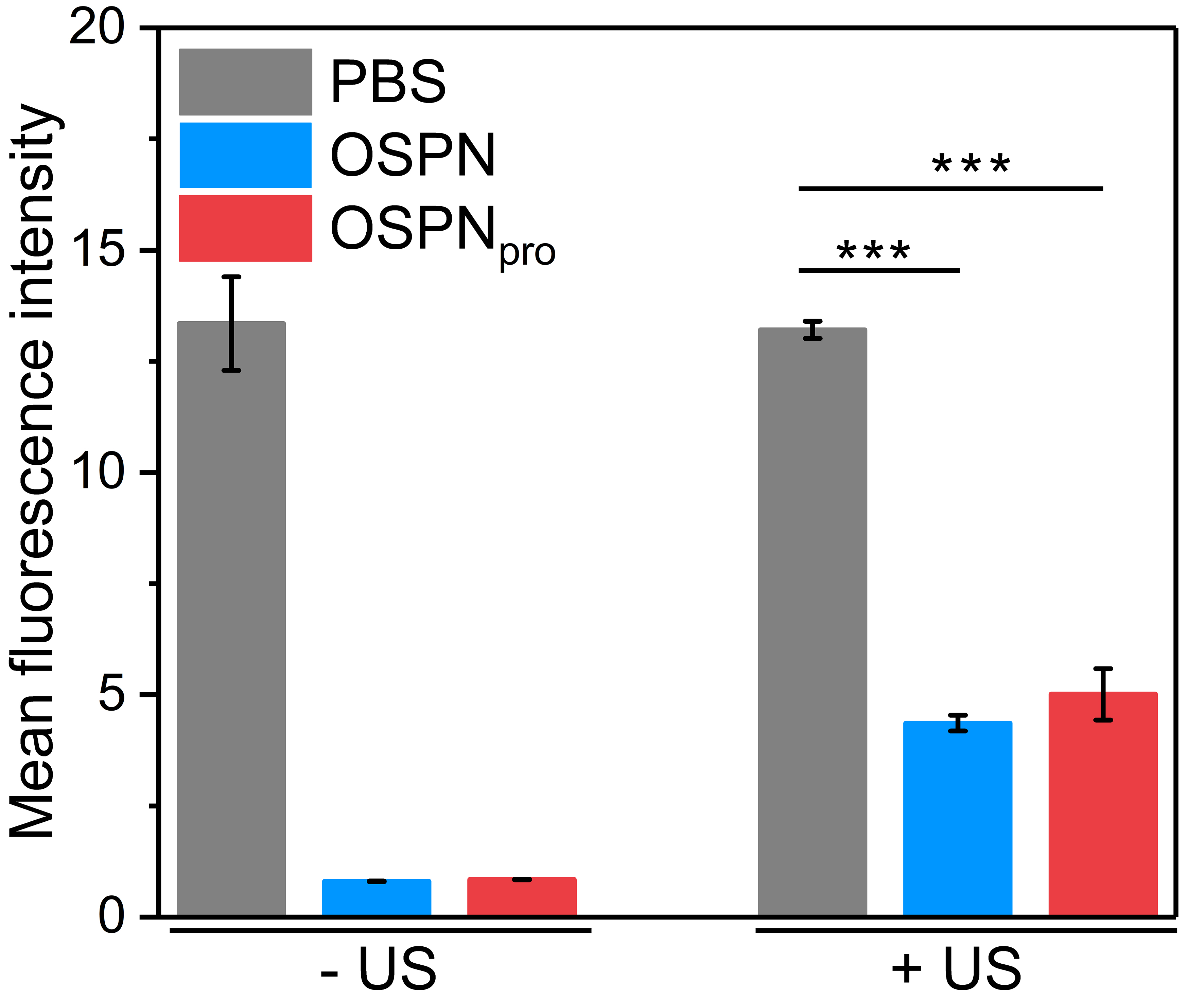
**

**Figure S14.** Mean fluorescence intensity of HIF-1α staining in 4T1 tumors after different treatments (n = 5). Mean ± SD are presented in data, two-tailed unpaired t test, *** p < 0.001.

**Figure S15.** Uncropped original images of Cleaved-caspase-3, GSDME-FL and GSDME-N in tumors via WB analysis.
